# BTB/POZ-MATH proteins regulate Arabidopsis seedling development by promoting auxin-independent degradation of the Aux/IAA protein IAA10

**DOI:** 10.1093/plphys/kiaf155

**Published:** 2025-04-21

**Authors:** Zhaonan Ban, Yueh-Ju Hou, Ellyse Ku, YingLin Zhu, Yun Hu, Natalie Karadanaian, Yunde Zhao, Mark Estelle

**Affiliations:** Department of Cell and Developmental Biology, School of Biological Sciences, University of California San Diego, La Jolla, CA, USA; Department of Cell and Developmental Biology, School of Biological Sciences, University of California San Diego, La Jolla, CA, USA; Department of Cell and Developmental Biology, School of Biological Sciences, University of California San Diego, La Jolla, CA, USA; Department of Cell and Developmental Biology, School of Biological Sciences, University of California San Diego, La Jolla, CA, USA; Department of Cell and Developmental Biology, School of Biological Sciences, University of California San Diego, La Jolla, CA, USA; Department of Cell and Developmental Biology, School of Biological Sciences, University of California San Diego, La Jolla, CA, USA; Department of Cell and Developmental Biology, School of Biological Sciences, University of California San Diego, La Jolla, CA, USA; Department of Cell and Developmental Biology, School of Biological Sciences, University of California San Diego, La Jolla, CA, USA

## Abstract

After germination, seedlings undergo etiolated development (skotomorphogenesis), enabling them to grow toward the soil surface. In Arabidopsis (*Arabidopsis thaliana*), etiolated seedlings exhibit rapid hypocotyl elongation, apical hook formation, and closed cotyledons to protect the meristem. In this study, we found that high-order mutants in the *BPM* (BTB/POZ-MATH) gene family displayed defects in seedling development, characterized by a shorter hypocotyl, early apical hook opening, and opened cotyledons in the dark. BPM1, BPM2, BPM4, and BPM5 exhibited distinct expression patterns and subcellular localization in etiolated seedlings. In a hypocotyl segment assay, the *bpm* mutants showed defects in auxin response, indicating impaired auxin signaling in the hypocotyl. Expression of the auxin reporter *DR5:GFP* was also altered in the *bpm1,4,5* mutant in various tissues compared with the wild type. Furthermore, yeast 2-hybrid, bimolecular fluorescence complementation, and co-immunoprecipitation assay analyses showed that BPM1 interacts with IAA10. Experiments in protoplasts indicated that BPM1 promotes IAA10 ubiquitylation and degradation, which was supported by greater IAA10 protein accumulation in the *bpm1,4,5* mutant background. In addition, IAA10 overexpression resulted in phenotypes similar to those of the *bpm* mutants, indicating that the BPMs may target the Aux/IAA proteins for ubiquitylation and degradation. Overall, our findings shed light on the key roles of the BPMs in auxin signaling during seedling development.

## Teaser

BPM (BTB/POZ-MATH domain-containing) proteins modulate auxin signaling via auxin-independent ubiquitylation and degradation of IAA10 (INDOLE ACETIC ACID–INDUCED PROTEIN10) during Arabidopsis seedling development.

## Introduction

Seedling emergence represents a pivotal phase in the plant life cycle, signifying the transition from a dormant seed to active growth. Following germination, seeds undergo 2 distinct developmental programs depending on whether they are growing in a dark or light environment. Etiolated development (skotomorphogenesis) occurs during seedling emergence in darkness, which is characterized in Arabidopsis by reduced root growth, elongated hypocotyls, apical hook formation, and closed cotyledons. During light-induced de-etiolation (photomorphogenesis), seedlings display reduced hypocotyl elongation and apical hook opening, along with cotyledon opening and expansion (Gendreau et al. 1997; Dong et al. 2019). Key components in light-responsive pathways and early signaling mechanisms have been identified to regulate seedling emergence. The E3 ubiquitin ligase COP1-based (CONSTITUTIVELY PHOTOMORPHOGENIC1) complex plays a central role in the light-dependent repression of photomorphogenesis in darkness (Huang et al. 2014; Hoecker 2017); PIF (PHYTOCHROME INTERACTING FACTOR) transcription factors are also crucial contributors to this process, since *pif* high-order mutants display constitutive photomorphogenic phenotypes in darkness (Leivar et al. 2008; Shin et al. 2009; Ni et al. 2017). Beyond light signaling, phytohormones also play key roles in seedling development, such as auxin (Chapman et al. 2012; Oh et al. 2014; Du et al. 2022), gibberellic acid (Alabadi et al. 2004; Achard et al. 2007; Feng et al. 2008; Chapman et al. 2012), brassinosteroid (Li and He 2016; Ravindran et al. 2021), ethylene (Zhong et al. 2014; Xiong et al. 2017), and jasmonate (Zheng et al. 2017). All these signals and their crosstalk regulate seedling morphogenesis (Chapman et al. 2012; Oh et al. 2014; Zhong et al. 2014; Xiong et al. 2017).

Auxin, a class of plant hormone, serves as a fundamental regulator of numerous physiological processes crucial for plant growth and development. At the cellular level, auxin regulates these processes through the control of cell division, expansion, and differentiation. The canonical auxin signaling pathway operates through the regulation of gene transcription mediated by SCF^TIR1/AFB^-Aux/IAA-ARF nuclear signaling modules. Three core components play vital roles in this pathway: the TIR1/AFB (TRANSPORT INHIBITOR RESPONSE/AUXIN SIGNALING F-BOX) co-receptors, Aux/IAA (INDOLE ACETIC ACID–INDUCED PROTEIN) transcriptional repressors, and ARF (AUXIN RESPONSE FACTOR) transcription factors. When auxin levels are low, the Aux/IAA proteins bind to ARFs and recruit transcriptional co-repressors TOPLESS (TPL), thereby preventing ARF activation of auxin responsive genes. When auxin levels are high, auxin promotes the binding of TIR1/AFBs to Aux/IAAs, facilitating the ubiquitylation and degradation of the Aux/IAAs. This, in turn, relieves ARF repression and activates the expression of auxin responsive genes (Lavy and Estelle 2016). There are 6 TIR1/AFBs, 29 Aux/IAAs, and 23 ARFs in Arabidopsis, and different modules provide the basis for diverse transcriptional outputs depending on various cellular and environmental contexts (Vernoux et al. 2011; Salehin et al. 2015). A previous study reported that auxin may regulate hypocotyl elongation by mediating the IAA3-ARF6/ARF8 module (Oh et al. 2014). During etiolated development, auxin promotes cell elongation in hypocotyl development by acid growth. Auxin induces the expression of *SAUR* genes through SCF^TIR1/AFB^ signaling, and the SAUR proteins repress PP2C.D phosphatase activity, preventing the dephosphorylation of PM H^+^-ATPase. As a result, the H^+^ pump remains in the activated state, leading to apoplast acidification and PM hyperpolarization, ultimately promoting cell expansion (Du et al. 2020). However, auxin inhibits cell elongation in apical hook development, indicating a biphasic control in etiolated seedling development, which may depend on auxin levels (Du et al. 2022). Recently, the TMK1-IAA32/IAA34 signaling pathway was reported to regulate apical hook development. The receptor-like kinase TMK1 was shown to stabilize IAA32 and IAA34, thereby mediating the inhibition of concave apical hook cells (Cao et al. 2019). In addition, auxin biosynthesis and transport also affect etiolated seedling phenotypes (Zhao et al. 2001; Vandenbussche et al. 2010).

The BPM (BTB/POZ-MATH) proteins belong to BTB/POZ protein family (BROAD COMPLEX, TRAMTRACK, and BRIC-A-BRAC/POZ and ZINC FINGER), which are substrate adaptors of Cullin3 E3 ligases. In Arabidopsis, there are 6 BPM proteins, named BPM1 to BPM6. BPMs feature a MATH domain within their *N*-terminal region responsible for binding substrates, and a BTB/POZ domain in their C-terminal region for binding CUL3a and CUL3b (Genschik et al. 2013; Ban and Estelle 2021). Previous studies have shown that BPMs may recognize a specific motif in their substrates, known as the SBC motif or SBC-like motif (Zhuang et al. 2009; Morimoto et al. 2017). The SBC motif is defined as a 5-residue ϕ-π-S-S/T-S/T, while the SBC-like motif is defined as ϕ-π-S-X-S/T, where ϕ represents a nonpolar residue, π represents a polar residue, and X can be any amino acid. BPMs interact with and regulate the turnover of various transcription factors, contributing to diverse roles in plant development (Weber and Hellmann 2009; Lechner et al. 2011; Chen et al. 2013, 2015; Morimoto et al. 2017; Julian et al. 2019; Mooney et al. 2019; Chico et al. 2020; Skiljaica et al. 2020). Several studies have highlighted the crucial role of BPMs in plant hormone signaling. In Arabidopsis, all 6 BPMs interact with HOMEOBOX 6 (HB6), a Class I homeodomain–leucine zipper transcription factor that functions as a negative regulator of abscisic acid (ABA) responses. The CUL3^BPM^ E3 ligase targets HB6 for ubiquitylation and degradation, affecting ABA responses (Lechner et al. 2011). BPM3 and BPM5 interact with multiple PP2CAs in the nucleus, promoting their ubiquitylation and turnover in an ABA-dependent manner (Julian et al. 2019). Recent findings revealed that CUL3^BPM^ targets MYC2/3/4, key transcriptional regulators of jasmonate response, for degradation. The stability of BPM3 is enhanced by jasmonate, indicating a negative feedback regulatory loop to modulate MYC levels and activities (Chico et al. 2020). Despite these findings, there is currently no evidence linking BPMs to auxin signaling.

Here, we show that high-order *bpm* mutants display defects in seedling development. Hypocotyl segment assays showed that auxin response is altered in the *bpm* mutants and changes in the activity of the *DR5:GFP* auxin reporter in various tissues in the mutants further indicate defects in auxin signaling. Moreover, we identified physical interactions between BPM1 and IAA10 through yeast 2-hybrid, bimolecular fluorescence complementation (BiFC) and co-immunoprecipitation (Co-IP) assays. Experiments also demonstrated that BPM1 increased the ubiquitylation level of IAA10 and decreased the stability of IAA10 when co-expressed in Arabidopsis protoplasts. There are also more IAA10 protein accumulation in *bpm1,4,5* mutant background. Additionally, IAA10 overexpression lines produced phenotypes similar to *bpm* mutants, suggesting that BPMs may act, at least in part, by targeting IAA10 protein for ubiquitylation and degradation. Overall, this study provides evidence that the BPMs play key roles in auxin signaling through IAA proteins.

## Results

### The *bpm* mutants exhibit defects in seedling development in the dark

**Figure 1.**
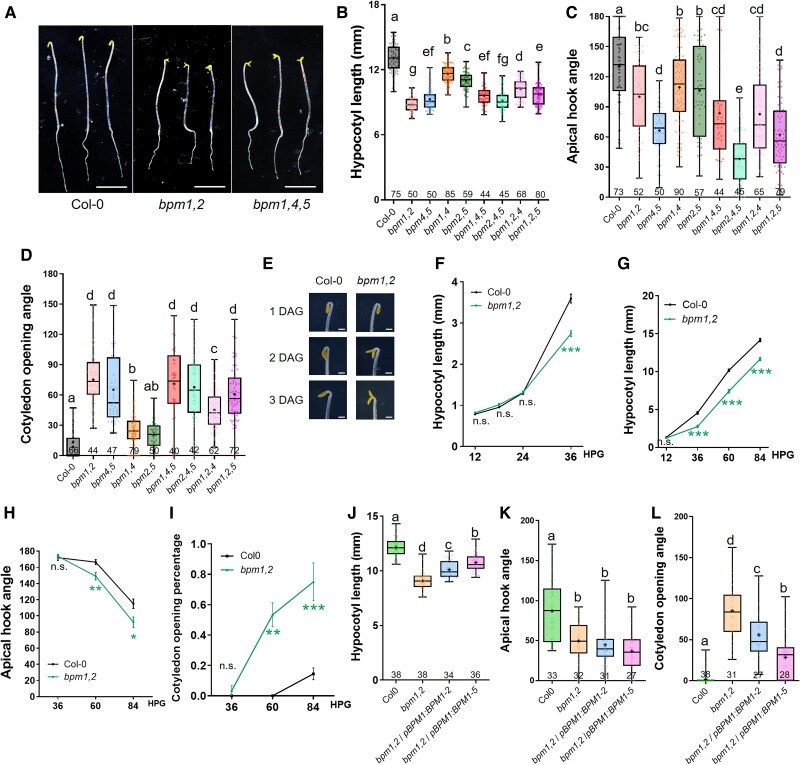
The *bpm* mutants exhibit defects in seedling development in the dark. **A)** 3DAG etiolated seedlings of Col-0, *bpm1,2*, and *bpm1,4,5* mutants. Scale bar is 5 mm. **B** to **D)** Quantification of defects in 3DAG *bpm* and Col-0 dark-grown seedings: **B)** hypocotyl length, **C)** apical hook angle, and **D)** cotyledon opening angle. **E)** Close-up of apical hook and cotyledon in Col-0 and *bpm1,2* at 1DAG to 3DAG time points. Scale bar is 1 mm. **F** to **I)** Quantification of hypocotyl length **(F**, **G)**, apical hook angle **(H)**, and cotyledon opening rate **(I)** at the indicated time points during etiolated seedling development in Col-0, and *bpm1,2*  **(F)** highlights the early elongation phase (first 24 h) and **(G)** focuses on the later elongation phase of the hypocotyl. About 25 seedlings were used in **(F)**, **(G)**, **(H)**, and **(I)**. Statistical differences according to one-way ANOVA analysis, *P < 0.05, **P < 0.01, ***P < 0.001. **J** to **L)** Complementation of *bpm1,2* by *pBPM1:BPM1-GFP*. Quantification of hypocotyl length **(J)**, apical hook angle **(K)**, and cotyledon opening angle **(L)** in Col-0, *bpm1,2*, and 2 independent *bpm1,2*/*pBPM1:BPM1-GFP* lines. Box plots in **(B** to **D)** and **(J** to **L)** represent the median and the first and third quartiles, with whiskers extending to minimum and maximum value; all data points are shown as dots. Different letters indicate statistical differences according to ordinary 1-way ANOVA coupled with Holm-Sidak's multiple comparison tests (*P* < 0.05), n.s. = not significant.

To explore the possible roles of the BPM proteins in seedling development, we attempted to generate deletion mutants of all 6 *BPM* genes (*BPM1*, *BPM2*, *BPM3*, *BPM4*, *BPM5*, and *BPM6*) using CRISPR-Cas9 technology. However, in the first round of transformants, we were unable to obtain effective knockout lines for *BPM3* and *BPM6*. To address this, we redesigned the primers and transformed new constructs, successfully obtaining *BPM3* and *BPM6* single mutant lines several months later than the other 4 *bpm* mutants. The 6 single mutants did not exhibit a visible phenotype compared with wild-type (WT; Col-0) plants, which is consistent with a previous report (Lechner et al. 2011). To overcome possible functional redundancy, we crossed the single mutants and generated several double and triple *bpm* mutant lines. Notably, due to the delayed generation of *bpm3* and *bpm6* mutants, their inclusion in high-order crosses was postponed. When phenotypes were identified in high-order mutants of *BPM1*, *BPM2*, *BPM4*, and *BPM5*, we prioritized investigating these phenotypes and decided not to pursue the generation of high-order mutants involving *BPM3* and *BPM6* at this time. We designated single mutants as *bpm1-2*, *bpm2-2*, *bpm4-2*, *bpm5-2* (Supplementary Fig. S1) and simplified *bpm1-2 bpm2-2* mutant to *bpm1,2* for brevity, and we applied similar abbreviations to other high-order mutants. In the first 2 generated mutants, *bpm1,2* and *bpm1,4,5*, we observed that they displayed shorter hypocotyls, and partially open apical hooks and cotyledons in 3-d-old etiolated seedlings (Fig. 1A). We then examined other high-order mutants of these *BPM* genes and found that all mutants examined showed similar defects but to varying extents (Fig. 1, B to D). When we checked the 4 single mutants, only *bpm1-2* and *bpm2-2* had slightly shorter etiolated hypocotyls than WT. No other significant differences in the single mutants compared with WT seedlings were observed in the dark (Supplementary Fig. S2). By analyzing the phenotypes of high-order mutants, we observed that BPM1 and BPM2 may play more important roles in hypocotyl elongation, since *bpm1,2* mutant showed the shortest hypocotyls. Regarding the apical hook phenotype, BPM4 and BPM5 may be more crucial, as the apical hooks of *bpm4,5* and *bpm2,4,5* were more open than the other lines. These results indicate that the *BPM* genes exhibit both functional redundancy and some specificity. To explore this idea further, we examined the phenotypes of *bpm1,2* at time intervals after germination in the dark (Fig. 1, E to I). The growth rate of WT seedlings was relatively slow up to 24 h postgermination (HPG) but then increased substantially during the 24HPG to 36HPG time interval. We found that *bpm1,2* exhibited a growth rate similar to WT in the early slow stage, but then was significantly slower in the rapid phase, indicating that BPM1 and BPM2 function in the rapid elongation stage (Fig. 1, F and G). Regarding the apical hook phenotype, *bpm1,2* showed normal hook formation at 36HPG, but displayed earlier opening of the apical hook compared with WT at later time points (Fig. 1H). The cotyledons of WT seedlings were closed in the dark, whereas the cotyledons of *bpm1,2* were open at later stages (Fig. 1I).

To confirm that these phenotypes were caused by loss of the *BPM* genes, we generated 2 independent *pBPM1:BPM1-GFP* lines in Col-0 background and crossed them with the *bpm1,2* mutant. Phenotypic analysis showed that the *pBPM1:BPM1-GFP* construct partially rescued the *bpm1,2* hypocotyl and cotyledon phenotype but not the defect in apical hook opening (Fig. 1, J to L), indicating that BPM1 plays more important roles in hypocotyl and cotyledon development compared with apical hook formation. The partially rescued phenotypes observed in *bpm1,2* double mutant by *BPM1* may also be attributed to the role of BPM2 in hook development. We also generated 2 independent 35S promoter-driven overexpression lines for each *BPM* gene in Col-0 background. Phenotypic analysis showed that one *35S:BPM1* line, two *35S:BPM2* lines, and one *35S:BPM5* line showed significantly longer hypocotyls than WT seedlings in the dark, while one *35S:BPM4* line showed shorter hypocotyl than WT, indicating that *BPM4* gene may play negative roles in hypocotyl elongation (Supplementary Fig. S3A). Regarding apical hook opening, two *35S:BPM4* lines displayed increased apical hook opening compared with Col-0 while all the other lines behaved similarly to the WT (Supplementary Fig. S3B). None of the *35S:BPM* overexpression lines had a cotyledon phenotype. We examined the transcript levels of different *BPM* genes in these overexpression lines (Supplementary Fig. S3C). According to our analysis, there is a clear correlation between expression levels and phenotypes in all of our lines, except for the *35S:BPM4-14* line. Combining all these phenotypes in the *bpm* mutants, *bpm1,2*/*pBPM1:BPM1-GFP* lines and *35S:BPM* lines, we showed that the *BPM* genes play important roles during etiolated development in seedlings. When we examined the light-grown *bpm* seedlings, we observed that *bpm1,2* and *bpm1,4,5* mutants had slightly longer hypocotyls compared with WT seedlings. This phenotype became more pronounced under short-day light conditions (Supplementary Fig. S4). Since this defect was quite modest, we did not pursue further studies of light-grown seedlings.

### Expression patterns of BPM1, BPM2, BPM4, and BPM5 in etiolated seedlings

**Figure 2.**
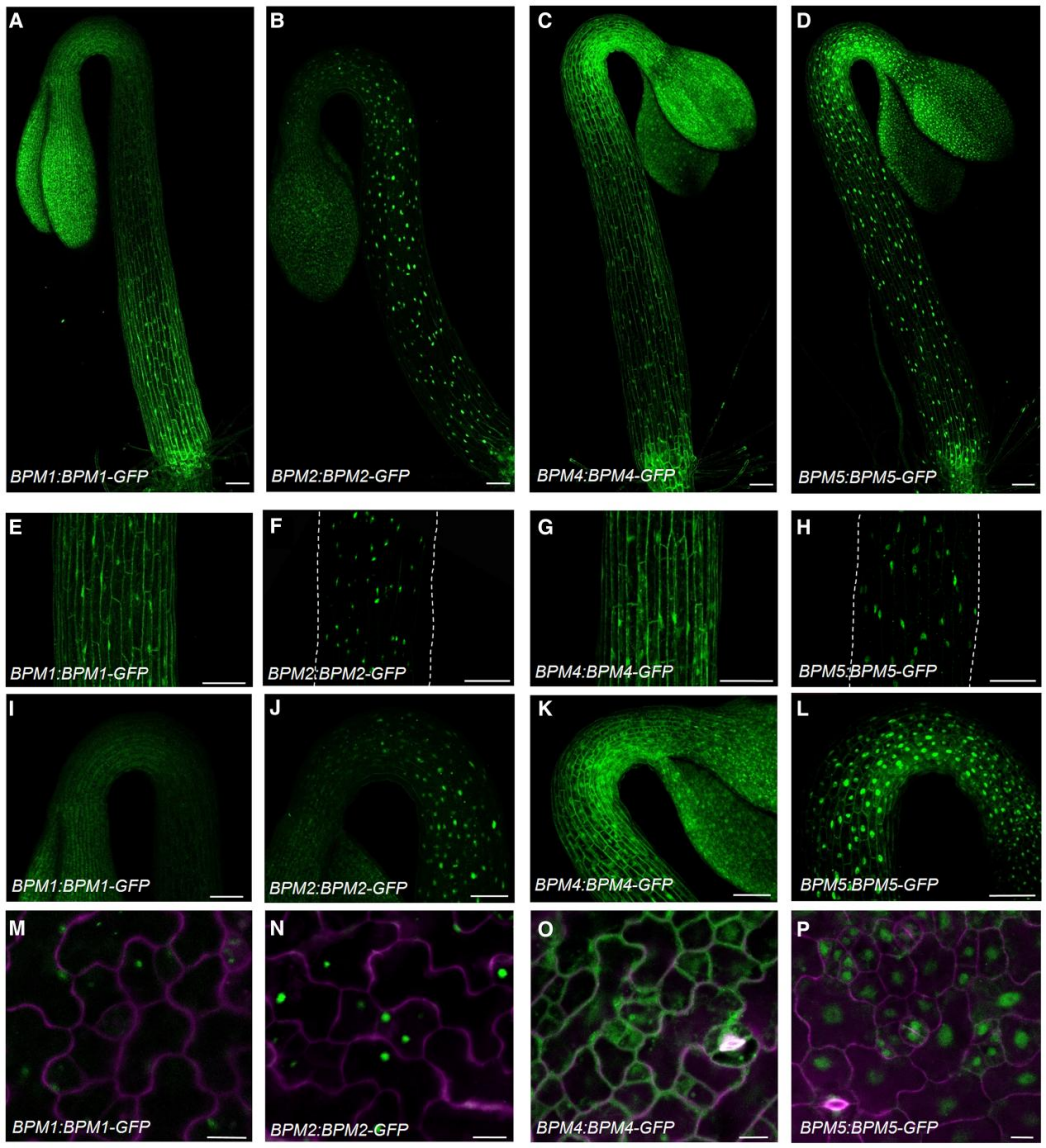
Expression pattern of *BPM1*, *BPM2*, *BPM4*, and *BPM5* genes in etiolated seedlings. GFP signal from native promoter-driven *BPM-GFP* lines showed different expression patterns. Whole etiolated seedlings **(A** to **D)**, hypocotyls **(E** to **H)**, apical hooks **(I** to **L)**, and cotyledon cells **(M** to **P)** from *pBPM1:BPM1-GFP*, *pBPM2:BPM2-GFP*, *pBPM4:BPM4-GFP*, and *pBPM5:BPM5-GFP* transgenic lines. The *pBPM2:BPM2-GFP* line was treated with 30 *µ*m Bortezomib for 6 h. Scale bars in **(A** to **L)** and **(M** to **P)** are 100 and 10 *µ*m, respectively.

Previous reports have shown that all 6 *BPM* genes are expressed broadly throughout the plant, albeit at different levels (Lechner et al. 2011). To further explore the expression of these genes in etiolated seedlings, we generated native promoter-driven transgenic lines of *BPM1*, *BPM2*, *BPM4*, and *BPM5* fused with *GFP*. Our findings indicated that all 4 genes are expressed in hypocotyls, apical hooks, and cotyledons with distinct patterns (Fig. 2). *BPM1* is expressed in the elongating hypocotyl, with weaker expression in the apical hook and cotyledon. The BPM1 protein was evident in the cytoplasm and the nucleus in these tissues. It is noteworthy that *BPM1* is not expressed in the early stages of etiolated seedling growth (before 24HPG). The pattern is also associated with the elongating part of hypocotyl, with higher expression in elongating cells than in other cells (Fig. 2, A, E, I, M). The *pBPM2:BPM2-GFP* transgenic line did not display any fluorescence, possibly due to a low expression level. Reasoning that BPM2 may be unstable, we then treated the seedlings with the proteasome inhibitor Bortezomib. After treatment with 30 *µ*m Bortezomib, BPM2 was clearly visible in hypocotyl and at very low levels in the apical hook and cotyledon (Fig. 2, B, F, J, N). Unlike BPM1, BPM2 is mostly localized to the nucleus. *BPM4* is highly expressed in the hypocotyl, apical hook, and cotyledon (Fig. 2, C, G, K, O), and the protein is localized to both cytoplasm and nucleus. It should be noted that there was a higher accumulation of BPM4 in the cytoplasm on the concave side of the apical hook. The *BPM5* gene is also highly expressed in the hypocotyl, apical hook, and cotyledon (Fig. 2, D, H, L, P). BPM5 accumulates mainly in the nucleus, with some proteins in the cytoplasm of apical hook cells.

Next, we determined whether BPM protein levels are regulated by light treatment. Western blot analysis using whole etiolated seedlings revealed that BPM4 and BPM5 levels were reduced after 6 h of white light, while BPM1 levels did not change (Supplementary Fig. S5, A to C). As stated above, BPM2-GFP was not visible without Bortezomib treatment. In contrast, the transcript levels of *BPM1*, *BPM2*, and *BPM4* increased 1.25-fold, 1.43-fold, and 1.33-fold after light treatment compared with control, respectively, while BPM5 levels remained unchanged (Supplementary Fig. S5H). These results indicate that light exerts a posttranscriptional effect on BPM4 and BPM5 levels. We also quantified the GFP signal in the hypocotyls and apical hooks of *pBPM4:BPM4-GFP* and *pBPM5:BPM5-GFP* lines and found that the levels of BPM4 and BPM5 decreased after 6 h of white light treatment (Supplementary Fig. S5, D and E). To explore the role of the proteasome in these changes, we treated seedlings with Bortezomib together with light and found that Bortezomib treatment increased BPM4/5 protein abundance, indicating that BPM4 and BPM5 are subject to degradation through the proteasome (Supplementary Fig. S5, F and G). These findings suggest that BPM proteins are degraded upon exposure to light, while transcript levels increase modestly.

To explore whether BPM levels are regulated by auxin, we treated *pBPM1:BPM1-GFP*, *pBPM4:BPM4-GFP*, and *pBPM5:BPM5-GFP* with 5 *µ*m IAA for 4 h. We found that BPM1 levels decreased by about 30% after this treatment, while BPM4 and BPM5 did not show significant differences compared with the no-treatment control (Supplementary Fig. S6, A to C). Analysis of transcript levels of the *BPM* genes following IAA treatment showed that *BPM1*, *BPM2*, and *BPM4* levels remained unchanged, with a slight increase in *BPM5* levels. This indicates that *BPM* genes are not significantly regulated by auxin treatment (Supplementary Fig. S6D).

### The *bpm* mutants have defects in auxin signaling

**Figure 3.**
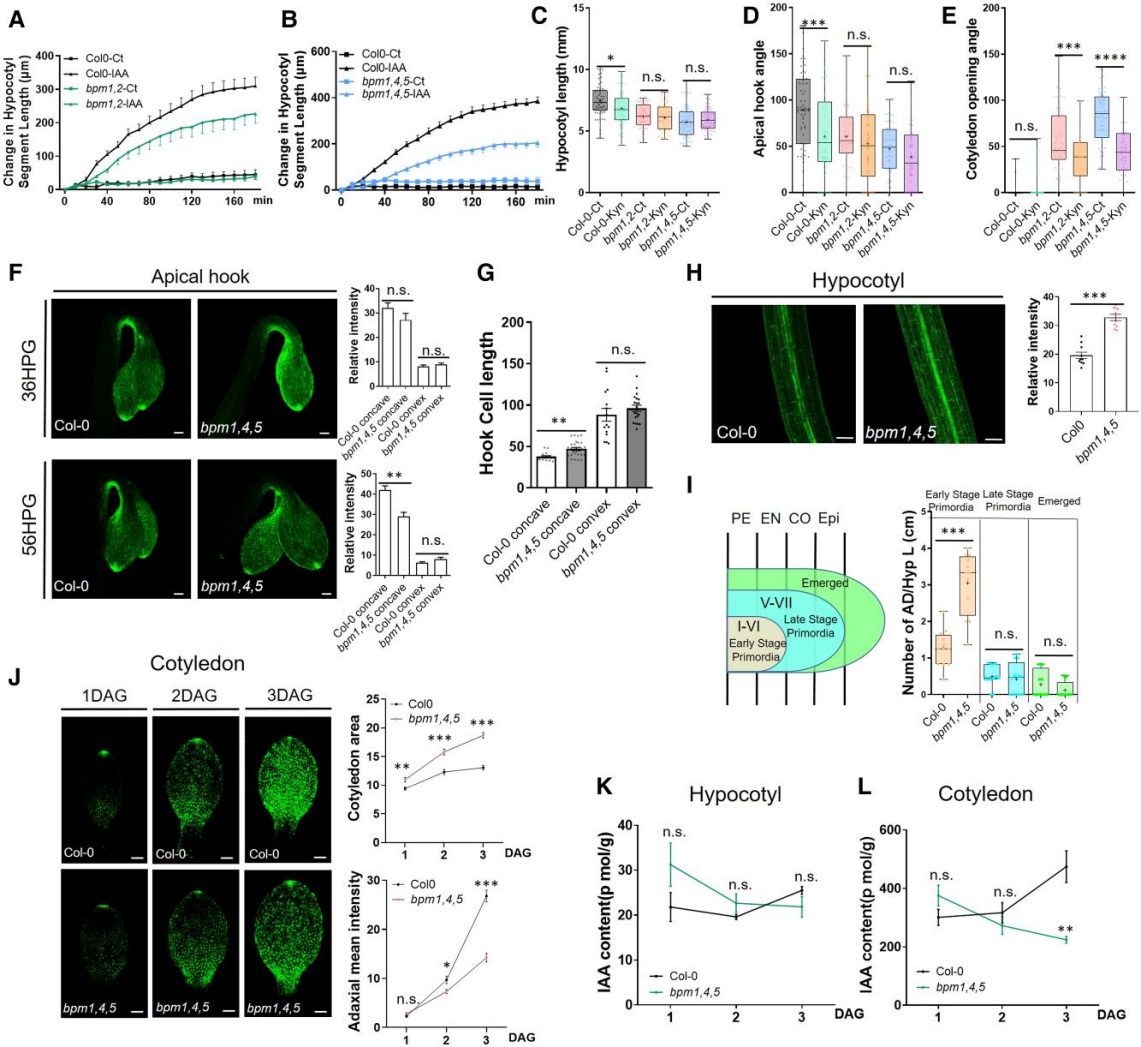
The *bpm* mutants have defects in auxin signaling. **A**, **B)** Hypocotyl segment elongation assay in response to 5 *µ*m IAA for 3 h in Col-0 and *bpm* mutants. About 10 seedlings in each line were used in these assays. **C** to **E)** Responses of Col-0, *bpm1,2*, and *bpm1,4,5* etiolated seedlings to treatment with the auxin synthesis inhibitor Kyn. Kyn (10 *µ*m) was used in this assay. 2DAG hypocotyls, 3DAG apical hooks, and cotyledons were used for the experiment. Fifty seedlings each genotype were used for quantification. **F)**  *DR5:GFP* signal in the apical hook region of Col-0 and *bpm1,4,5* seedlings at 36HPG and 56HPG. Quantification results are shown on the right side. Scale bars = 100 *µ*m. Six seedlings in each genotype were used for quantification. **G)** Cell length in apical hook regions of Col-0 and *bpm1,4,5* seedlings on concave and convex sides. About 20 cells in each genotype were used for quantification. **H)**  *DR5:GFP* signal in 2DAG hypocotyls of Col-0 and *bpm1,4,5* seedlings. Quantification is shown on the right side. Scale bar = 100 *µ*m. Ten seedlings in each genotype were used for quantification. **I)** Hypocotyl adventitious root primordia and emerged hypocotyl adventitious roots in Col-0 and *bpm1,4,5*. Twelve seedlings for each genotype were used. PE, pericycle; EN, endodermis; CO, cotex; Epi, epidermis. **J)**  *DR5:GFP* signal on the adaxial side of cotyledons in Col-0 and *bpm1,4,5* at 1DAG to 3DAG. Scale bar = 100 *µ*m. Cotyledon area and GFP intensity were measured and shown to the right of the images. About 15 cotyledons for each genotype were used. **K**, **L)** IAA content was measured in hypocotyls **(K)** and cotyledons **(L)** of Col-0 and *bpm1,4,5* etiolated seedlings at 1DAG, 2DAG, and 3DAG. Five biological replicates were used in this assay. Box plots in **(C** to **E)** and **(I)** represent the median and the first and third quartiles, with whiskers extending to minimum and maximum value; all data points are shown as dots. Statistical differences in these assays are according to Student's *T*-test analysis, *P < 0.05, **P < 0.01, ***P < 0.001. Error bars indicate ±SEM.

To assess potential defects in auxin response in the *bpm* mutants given their reduced hypocotyl length, we examined the growth response of hypocotyl segments to 5 *μ*m IAA and NAA. Dissected hypocotyl segments from etiolated seedlings were treated with compounds, and imaged every 10 min for 3 h. We found that both *bpm1,2* and *bpm1,4.5* were resistant to IAA and NAA compared with the WT, indicating that these *BPM* genes are required for auxin-dependent growth during hypocotyl elongation (Fig. 3, A and B; Supplementary Fig. S7, A and B). We also investigated how WT and the *bpm* mutants respond to the auxin synthesis inhibitor, L-kynurenine (Kyn). WT plants exhibited reduced hypocotyl growth and a more open apical hook when treated with 10 *µ*m Kyn. In contrast, the *bpm* mutants showed resistance to Kyn treatment in both hypocotyl and apical hook development (Fig. 3, C and D). Interestingly, in WT seedlings, cotyledons remained closed after Kyn treatment. In the *bpm1,2* and *bpm1,4,5* mutants, Kyn significantly inhibited the opening of cotyledons, indicating that abnormal cotyledon opening in the mutants depends on auxin (Fig. 3E). Because we observed defects in auxin response in *bpm* etiolated seedlings, we determined the transcript levels of auxin biosynthesis genes *YUCCA1*, *YUCCA2*, and *YUCCA3* and the auxin transport genes *AUX1* and *LAX3*. We found that these genes are downregulated in *bpm1,4,5,* with *YUCCA2/3* and *LAX3* levels at about 50% percent compared with WT (Supplementary Fig. S8, A and B). Several *SAUR* genes, which promote cell elongation during hypocotyl elongation and regulate cell expansion during cotyledon opening, were also assessed in both hypocotyls and cotyledons. Results showed that *SAUR14*/*SAUR19*/*SAUR22*/*SAUR65* were downregulated in hypocotyls but upregulated in cotyledons, which suggests that expression of these genes is inhibited in hypocotyl and enhanced in cotyledon (Supplementary Fig. S8C). This is consistent with the *bpm1,4,5* phenotypes of shorter hypocotyl and opened cotyledon.

To visualize auxin signaling in the *bpm* mutants, we generated the *bpm1,4,5 DR5:GFP* line and examined GFP signal in the hypocotyl, apical hook, and cotyledon. At 36HPG, we did not see a difference between the WT and *bpm1,4,5* in the apical hook, while at 56HPG, the GFP signal was significantly decreased on the concave side of *bpm1,4,5* plants compared with WT, correlating with the apical hook opening phenotype of the *bpm1,4,5* mutant (Fig. 3F). We also quantified the epidermal cell length in the hook region of WT and *bpm1,4,5* seedlings. The cell length on the concave side of *bpm1,4,5* was longer than in WT, while there was no difference in the convex side. These results indicate that there is reduced auxin signaling on the concave side of apical hook in *bpm1,4,5* seedlings, resulting in the failure to maintain the hook (Fig. 3G). In the hypocotyl, GFP signal accumulates in the stele region. Surprisingly, we found that the GFP signal in the stele region was stronger in *bpm1,4,5* than in WT at 2 d after germination (DAG) (Fig. 3H). Subsequently, we carefully examined adventitious root development in the hypocotyl region and observed a higher number of early-stage adventitious root primordia in the hypocotyl of *bpm1,4,5* seedlings compared with the WT. No significant differences were detected in late-stage primordia and emerged adventitious roots between *bpm1,4,5* and WT seedlings (Fig. 3I). Interestingly, when we examined lateral root development in light-grown seedlings, we also observed a higher number of early-stage lateral root primordia in *bpm* mutants compared with the WT, but no differences in other stages of lateral root development (Supplementary Fig. S9). These results suggest that BPMs negatively regulate adventitious roots and lateral root initiation. When we measured the amount of auxin in the hypocotyl of etiolated seedlings, there were no differences in auxin levels between the WT and the *bpm1,4,5* mutant at all 3 time points, suggesting that the phenotype in the hypocotyl is caused by changes in auxin response (Fig. 3K).

In the cotyledon, we quantified cotyledon area and *DR5:GFP* signal on the adaxial side at 1DAG to 3DAG (Fig. 3J). At 1DAG, the area of *bpm1,4,5* was 16.9% larger than WT. Along with cotyledon opening at 2DAG and 3DAG, the *bpm1,4,5* cotyledon area also rapidly increased, indicating rapid cell expansion. In contrast, the cotyledon area of WT increased very slowly. There were 28.3% and 43.1% differences between *bpm1,4,5* and WT in cotyledon area at 2DAG and 3DAG, respectively. The *DR5:GFP* signal was at a similar level at 1DAG between *bpm1,4,5* and WT; interestingly, GFP levels increased significantly at 2DAG and 3DAG in WT but increased very slowly in *bpm1,4,5*. We also found that IAA levels increased in the cotyledons of WT seedlings, but decreased in *bpm1,4,5* mutant, resulting in a significant difference in IAA levels at 3DAG between the WT and *bpm1,4,5* line (Fig. 3L). Combined with the Kyn treatment analysis, these results suggest that cotyledon development relies on an appropriate amount of auxin, as too low or too high amounts suppress cotyledon opening and expansion.

### BPM proteins interact with IAA6/10/11 proteins

**Figure 4.**
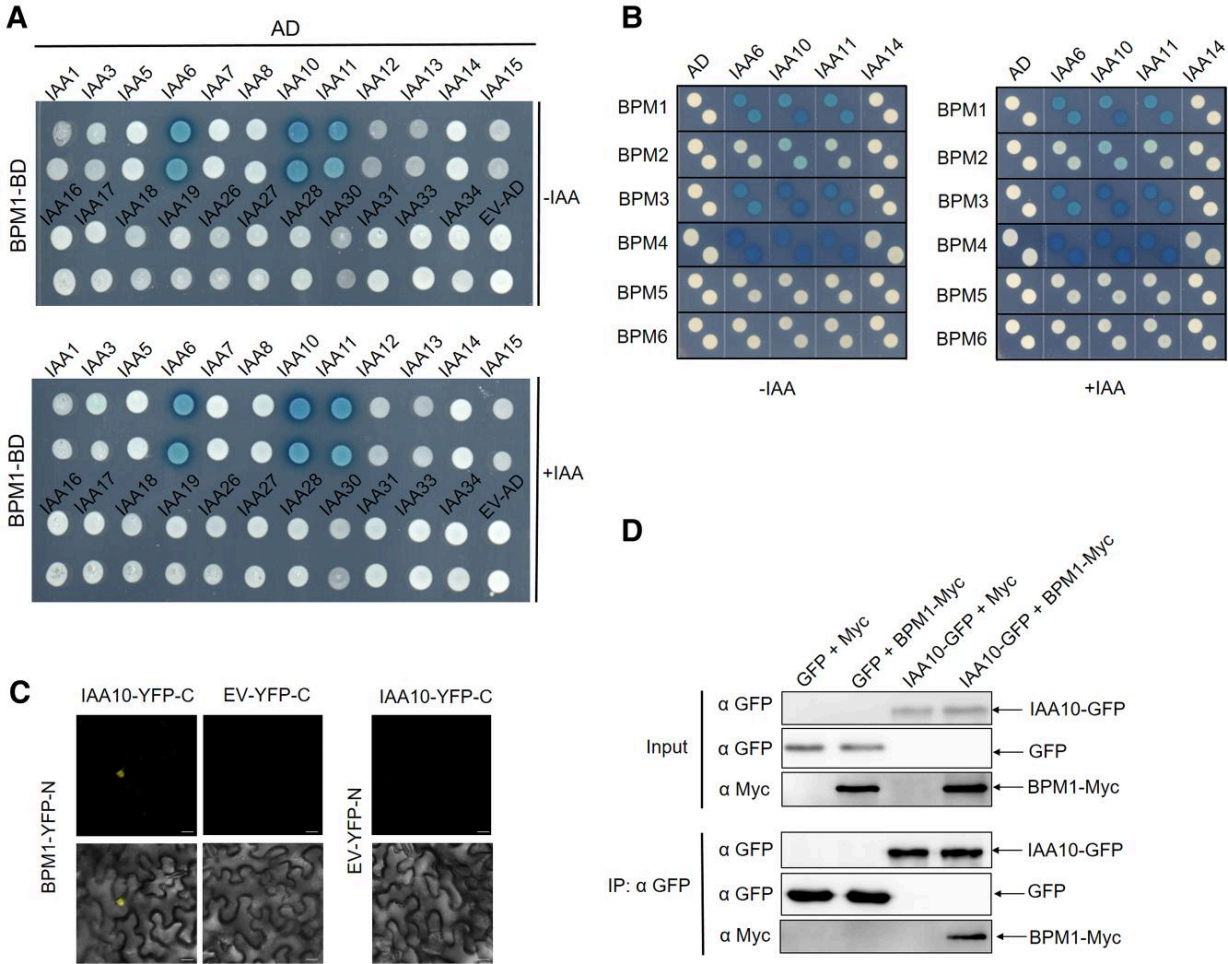
BPMs interact with Aux/IAA proteins. **A)** Yeast 2-hybrid test of BPM1 with Aux/IAA proteins. **B)** Yeast 2-hybrid test of 6 BPM proteins with IAA6/10/11/14. IAA (50 *µ*m) was used in these assays. **C)** Direct interaction between BPM1 and IAA10 in *N. benthamiana* leaves. Confocal images of YFP fluorescence in leaves co-infiltrated with agrobacteria containing BPM1-YFP(N) and IAA10-YFP(C), BPM1-YFP(N) and EV-YFP(C), or EV-YFP(N) and IAA10-YFP(C). Scale bars = 20 *µ*m. **D)** Co-IP of Myc-tagged BPM1 and GFP-tagged IAA10 using anti-Myc and anti-GFP antibodies. Total protein extracts were prepared from Arabidopsis mesophyll protoplasts transfected with plasmids expressing *GFP* and *Myc*, *GFP* and *BPM1-Myc*, *IAA10-GFP* and *Myc*, and *IAA10-GFP* and *BPM1-Myc*.

As BPM proteins function as substrate adaptors in CUL3-E3 ligases, we hypothesized that they may regulate the stability of Aux/IAA proteins, thereby modulating auxin signaling. To test this hypothesis, we used the yeast 2-hybrid system to probe for interactions between BPM1 and Aux/IAA proteins. Among the 23 Aux/IAA proteins we tested, we found that BPM1 interacts with IAA6, IAA10, and IAA11 with or without auxin supplementation, but not with the other Aux/IAA proteins (Fig. 4A, Supplementary Fig. S10). Next, we tested whether other BPMs also interact with IAA6/10/11 in the yeast 2-hybrid system. We found that BPM3 and BPM4 also strongly interact with IAA6/10/11, and BPM2 has a weak interaction with IAA10, regardless of the presence of IAA. BPM5 and BPM6 do not interact with these IAA proteins (Fig. 4B). Because an interaction between IAA10 and BPM3 had been reported in an Arabidopsis interactome study (Arabidopsis Interactome Mapping Consortium 2011), we focused on IAA10 as a representative. We further confirmed the interaction between BPM1 and IAA10 using BiFC assay in *Nicotiana benthamiana* leaves and Co-IP assay in Arabidopsis mesophyll protoplasts (Fig. 4, C and D). Since BPM proteins were reported to recognize an SBC motif in substrate proteins, we searched for such a motif in the IAA10 sequence and identified the LSSSS sequence between the EAR domain and DII degron (Supplementary Fig. S11A). To investigate the function of this motif, we generated a mutant version of IAA10 with a substitution in the DII degron (GWP**S**L) and a version of IAA10 with substitutions in the SBC domain (LS**AAA)**, and assessed their interactions with BPM1. Yeast 2-hybrid analysis showed that IAA10 (WT) and IAA10 (DII) interact with BPM1 with or without IAA supplementation, whereas IAA10 (SBC) did not interact with BPM1 in the presence or absence of IAA. TIR1 interacted with IAA10 (WT) and IAA10 (SBC) only in the presence of IAA, while it did not interact with IAA10 (DII) under either condition as expected (Supplementary Fig. S11B).

### BPM1 regulates the ubiquitylation and degradation of IAA10

**Figure 5.**
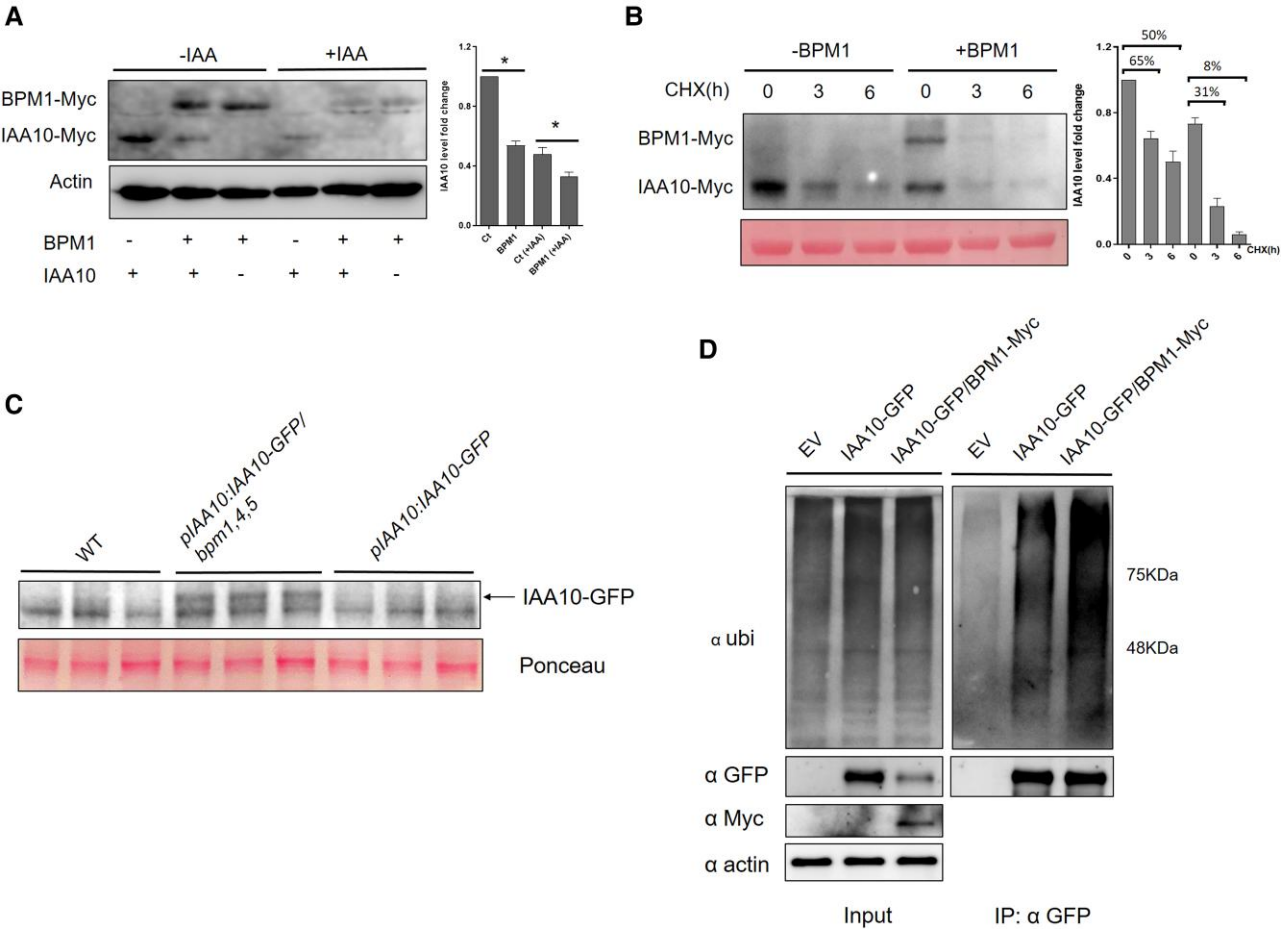
BPM1 regulates the stability of IAA10 protein. **A)** Degradation assay in Arabidopsis protoplasts. IAA10 levels were quantified with or without addition of BPM1. Protein levels of IAA10 were normalized to actin (Student's *T*-test, *P < 0.05). **B)** Degradation rate of IAA10 in protoplasts. After transformation and incubation overnight, protoplasts were treated with 50 *μ*m CHX, and protein levels were analyzed at the indicated times. Error bars in **(A)** and **(B)** indicate ±SEM. **C)** Western blot analysis of IAA10-GFP in *pIAA10:IAA10-GFP* and *pIAA10:IAA10-GFP/bpm1,4,5* lines. Seedlings were treated with 5 *µ*m IAA overnight to increase transcription. Hypocotyls of etiolated seedlings from each line were dissected for protein extraction, with 3 biological replicates shown in the figure. **D)** Detection of ubiquitylated IAA10 levels with or without co-expression of BPM1 in Arabidopsis protoplasts. Total protein extracts were used as input to detect overall ubiquitylated proteins, IAA10-GFP, and BPM1-Myc. Total proteins were precipitated using GFP-Trap magnetic beads, and protein loading was adjusted to obtain comparable amounts of the IAA10-GFP bands. Three biological replicates were performed in these assays.

To further explore the impact of BPM1 on IAA10 stability, we expressed BPM1-Myc and IAA10-Myc in Arabidopsis mesophyll protoplasts. Co-expression of BPM1-Myc together with IAA10-Myc resulted in decreased accumulation of IAA10-Myc compared with expression of IAA10-Myc alone, indicating that BPM1 regulates the level of IAA10. We also treated the protoplasts with IAA and found that the overall levels of IAA10 were reduced without BPM1 present, presumably due to TIR1/AFB-mediated degradation. However, we also found that co-expression of BPM1-Myc with IAA10-Myc resulted in a further decrease in IAA10 levels, suggesting that BPM1 regulates IAA10 levels independently of auxin (Fig. 5A). This is consistent with our observation that the interaction between BPM1 and IAA10 in the Y2H assay is not affected by auxin. We also constructed an SBC mutant version of IAA10-Myc and tested its degradation in a protoplast assay. First, we found that the IAA10 (SBC) mutant version accumulates more protein compared with the IAA10 (WT) version. When co-expressed with BPM1, the protein level of IAA10 (WT) decreased substantially, while the IAA10 (SBC) also decreased but to a lesser extent (Supplementary Fig. S11C). We then analyzed the degradation rate of IAA10 in protoplasts by treating them with cycloheximide (CHX), a protein synthesis inhibitor. Protoplasts were prepared to express *IAA10-Myc* with or without *BPM1-Myc*. The results showed that IAA10 protein was degraded over time following CHX treatment, and the degradation occurred much more rapidly when BPM1 was co-expressed (Fig. 5B).

To investigate the impact of BPMs on IAA10 protein levels in vivo, we initially crossed the *35S:IAA10* line with *bpm1,4,5* mutant. However, *IAA10* gene expression was silenced in the crossed line, likely due to excessive IAA10 protein accumulation. We then generated a *pIAA10:IAA10-GFP* line and crossed it with *bpm1,4,5* mutant. Given that Aux/IAA proteins are short-lived and turnover quickly, IAA10 protein levels were very low, making detection difficult under normal conditions in both WT and *bpm1,4,5* backgrounds. Upon IAA treatment to enhance transcription, we observed that IAA10-GFP remained faint in the WT background but was detectable in the *bpm1,4,5* background, indicating increased IAA10 protein accumulation in *bpm1,4,5* (Fig. 5C). To further test whether BPM1 regulates IAA10 protein stability via ubiquitylation, we expressed *IAA10-GFP* with or without *BPM1-Myc* in protoplasts, and we then used GFP-Trap beads to precipitate the IAA10-GFP protein and examined its ubiquitylated levels. Results showed that co-expression with BPM1 led to a higher level of ubiquitylation on IAA10-GFP than expression of IAA10-GFP alone, suggesting that BPM1 may target IAA10 for ubiquitylation (Fig. 5D). All together, these findings imply that BPMs function as substrate adaptors of CUL3-E3 ligase and regulate the ubiquitylation and degradation of IAA10 protein, subsequently influencing auxin signaling pathway.

### BPMs may recognize the IAA10 protein as a target during seedling development

**Figure 6.**
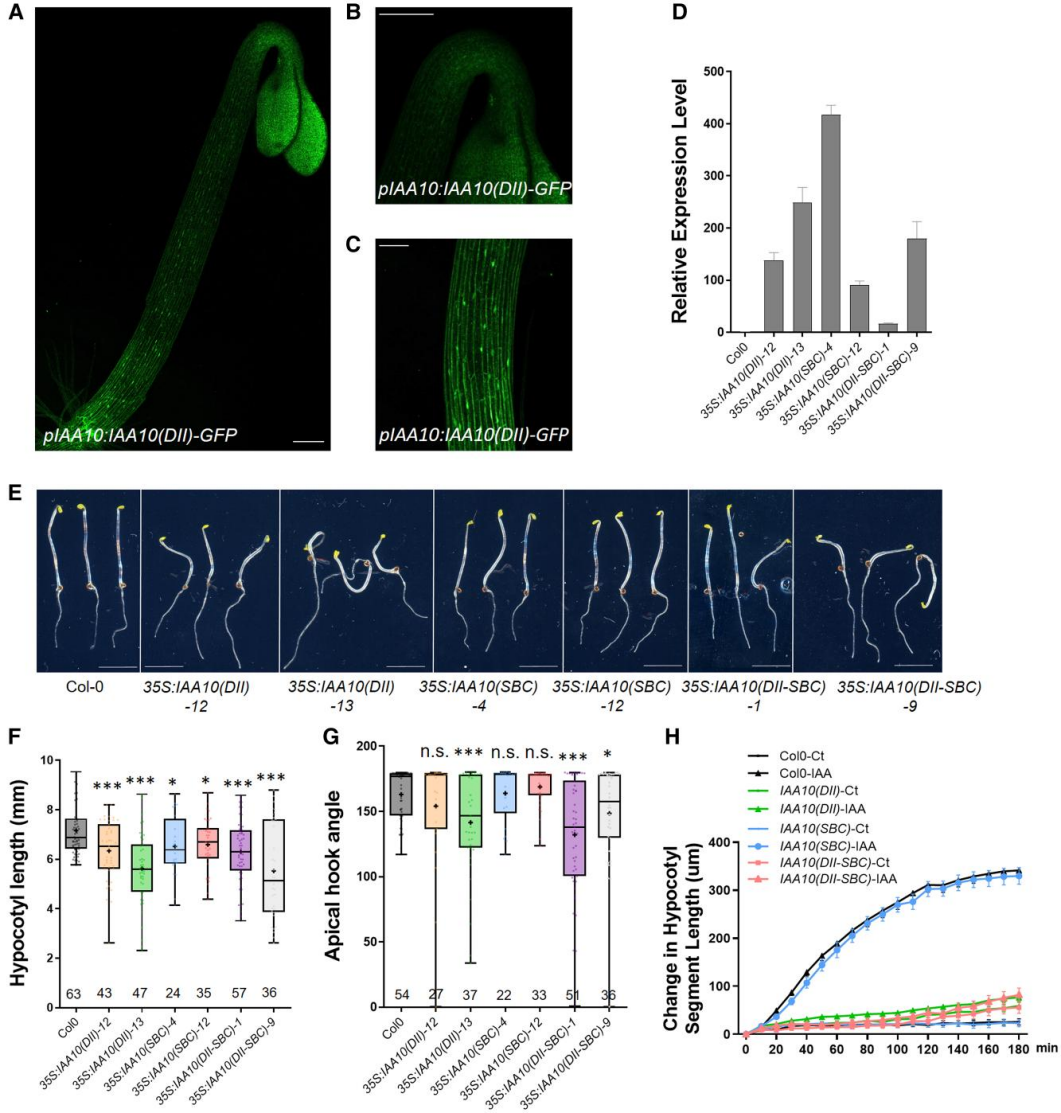
IAA10 negatively regulates hypocotyl growth and apical hook formation in the dark. **A)** GFP signal from *pIAA10:IAA10-GFP* line showed the expression pattern of the *IAA10* gene. Whole etiolated seedling **(A)**, apical hook **(B)**, and hypocotyl **(C)**. Scale bars = 100 *µ*m. **D)**  *IAA10* transcript levels in different versions of *35S:IAA10* lines. Ten seedlings in each line were used in this assay. Data are represented as mean ± SEM. **E)** 2DAG etiolated seedlings of Col-0, *35S:IAA10(DII)-12*, *35S:IAA10(DII)-13*, *35S:IAA10(SBC)-4*, *35S:IAA10(SBC)-12*, *35S:IAA10(DII-SBC)-1*, and *35S:IAA10(DII-SBC)-9*. Scale bar = 5 mm. **F**, **G)** Quantification of hypocotyl and apical hook angles in *35S:IAA10* lines compared with Col-0. Box plots in **(F**, **G)** represent the median and the first and third quartiles, with whiskers extending to the minimum and maximum value; all data points are shown as dots. Statistical differences according to Student's *T*-test analysis by comparing each genotype to Col-0. *P < 0.05, **P < 0.01, ***P < 0.001. **H)** Hypocotyl segment elongation in response to 5 *µ*m IAA in Col-0 and *35S:IAA10* lines. About 15 seedlings in each line were used in this assay. Error bar indicates ±SEM.

Since our data suggest that IAA10 is a target of the BPMs, we checked the expression pattern of *IAA10* in the *pIAA10:IAA10-GFP* line. We found that the *IAA10* gene is expressed in etiolated seedlings, and the IAA10 protein accumulates in the nucleus and cytoplasm, similar to BPM1 (Fig. 6, A to C). We also generated DII, SBC, DII-SBC mutant versions of *35S:IAA10* overexpression lines and examined their phenotypes (Fig. 6, D to G). The results showed that overexpression of *IAA10 (SBC)* results in mild defects in hypocotyl elongation, but not apical hook. *35S:IAA10 (DII)* and *35S:IAA10 (DII-SBC)* line phenotypes are similar to the *bpm1,4,5* line, with shorter hypocotyls (Fig. 6F) and apical hook defects (Fig. 6G). Some etiolated seedlings from stronger *IAA10 (DII)* and *IAA10 (DII-SBC)* lines also displayed partially opened cotyledons and agravitropic hypocotyls and roots. When comparing *IAA10 (DII)* with *IAA10 (DII-SBC)* lines phenotypes, they did not show very significant differences. We conducted the hypocotyl segment assay using different *35S:IAA10* lines, and found *IAA10 (DII)* and *IAA10 (DII-SBC)* lines exhibited resistance to IAA treatment in hypocotyl elongation compared with WT, whereas *IAA10 (SBC)* line did not show a significant difference (Fig. 6H). These results suggest that BPMs may recognize the SBC domain of IAA10 leading to degradation, contributing to etiolated seedling development. However, the mild phenotypes observed in *IAA10 (SBC)* line also suggest that BPMs may target other Aux/IAA proteins or other transcription factors during seedling development.

## Discussion

Seedling emergence is a complicated process in which both light and plant hormones play vital roles (Feng et al. 2008; Huang et al. 2014; Pham et al. 2018; Krahmer and Fankhauser 2024). During skotomorphogenesis, auxin acts to regulate cell expansion through the TIR1/AFB-Aux/IAA pathway. Expansion growth occurs when auxin levels increase, resulting in degradation of the Aux/IAA proteins and increased expression of auxin-regulated genes, including the *SAUR* genes (Du et al. 2020). Although the SCF^TIR1/AFB^ E3 ligases are major regulators of Aux/IAA degradation, it is possible that other E3 ligases may also play a role. Here, we show that members of the BPM family of proteins, substrate adapters for CRL3 E3 ligases, function in auxin signaling during etiolated seedling development. We also present evidence that BPMs may function by regulating the stability of the Aux/IAA proteins, with a particular focus on IAA10 (Supplementary Fig. S12). Regarding the ARF partners of IAA10, interactome mapping and protein network studies suggest that ARF2, ARF9, ARF18, and ARF19 are potential interactors of IAA10. These ARFs have been reported to regulate hypocotyl (Li et al. 2004), lateral root and adventitious root development (Okushima et al. 2007; Lee et al. 2009), making them strong candidates for involvement in potential BPMs-IAA10-ARFs modules that regulate seedling development.

There are 6 members in the *BPM* gene family. Previous results, as well as our studies, did not reveal a phenotype in the single *bpm* mutants suggesting that these genes have redundant functions. Here, we use CRISPR-Cas9 technology to generate new loss-of-function alleles of *BPM1*, *BPM2*, *BPM3*, *BPM4*, *BPM5*, and *BPM6*. By constructing double and triple mutants, we demonstrated that these genes function in various aspects of etiolated seedling development, including hypocotyl elongation, apical hook opening, and cotyledon expansion. However, individual members of the family contribute to these processes to different degrees. BPM1 and BPM2 have important roles in hypocotyl elongation, while BPM4 and BPM5 function more in apical hook maintenance. It is noteworthy that although *bpm3* and *bpm6* were not included in our high-order mutants due to the delayed generation of *bpm3* and *bpm6* single mutants, BPM3 and BPM6 are likely to play roles in this context. The phenotypic analysis is also consistent with *BPM* expression patterns in different tissues. We observed *BPM1* and *BPM2* are more highly expressed in the hypocotyl region, while *BPM4* and *BPM5* are strongly expressed in the apical hook region. Interestingly, these 4 BPMs showed distinct subcellular localization patterns. BPM2 and BPM5 mainly accumulate in the nucleus, while BPM1 and BPM4 accumulate in both nucleus and cytoplasm. The TIR1/AFBs auxin receptors also partition differentially between the nucleus and cytoplasm. Strikingly, TIR1 is primarily expressed in nuclei, while AFB1 is primarily in the cytoplasm (Prigge et al. 2020; Chen et al. 2023). Recently, it was reported that cytoplasmic AFB1 functions in a nontranscriptional rapid auxin response (Prigge et al. 2020; Serre et al. 2021). In addition, AFB1 may have an inhibitory role in canonical auxin signaling (Dubey et al. 2023). It is reasonable to hypothesize that the localization of different BPM proteins may contribute to their functions. We also noticed that BPM4 may play negative roles in seedling development based on the phenotypes of *35S:BPM4* line phenotypes. It will be interesting to explore how the localization of the BPM proteins relates to their functions.

According to our results, in the dark, BPMs positively regulate cell elongation in the hypocotyl, but negatively regulate cell elongation on the concave side of the apical hook during opening. During cotyledon development, the BPMs negatively regulate cotyledon opening and expansion. Interestingly, we also found that there are more adventitious root primordia in the hypocotyls of the *bpm1,4,5* mutant, indicating BPMs negatively regulate adventitious root formation. Similarly, BPMs negatively regulate the formation of lateral roots under light conditions. Combining these observations, it appears that auxin signaling is substantially mis-regulated in the *bpm* mutants. Since we found that several BPM proteins interact with IAA10, and BPM1 regulates the stability of IAA10, it is possible that the auxin defects are related to increased levels of this Aux/IAA protein. Consistent with this, we found that overexpression of *IAA10* resulted in a phenotype similar to that of the *bpm* mutants. However, *IAA10-SBC* overexpression lines did not show strong hypocotyl defects or produce a cotyledon phenotype. This may be due to stabilization of a different Aux/IAA protein. It is also well known that the BPMs function in a number of signaling pathways by targeting various substrates for degradation, and it is possible that the defect in cotyledon development may be related to a different BPM target.

Interestingly, we also found that auxin levels are altered in the cotyledons but not the hypocotyls of the *bpm* mutants. In the cotyledons of WT seedlings, auxin levels increase during seedling development, while in the *bpm* mutants, they decrease. These results indicate that the BPMs may regulate auxin signaling, as well as auxin biosynthesis, metabolism, or transport, either directly or indirectly in cotyledon. In support of this idea, we found that *YUCCA* and *LAX* genes are downregulated in *bpm1,4,5* mutant. Because of the complexity of the auxin regulatory system, with interdependent regulation of signaling, transport, and metabolism, it is possible that the BPMs affect auxin signaling both directly and indirectly.

In the canonical auxin signaling pathway, auxin acts to promote the interaction between the TIR1/AFBs and Aux/IAA proteins. In contrast, our results indicate that auxin does not contribute to the interaction between the BPMs and the Aux/IAA proteins. The TIR1/AFB proteins recognize the DII degron of Aux/IAAs and regulate their ubiquitylation and degradation. According to our results, the BPMs may recognize the SBC motif of the Aux/IAAs and regulate their ubiquitylation and degradation. BPM1 reduces IAA10 stability without IAA treatment, indicating BPMs may constitutively function in the auxin pathway. We also found that BPM1 level decreased slightly with auxin treatment, while BPM4 and BPM5 were not regulated by auxin, strengthening this hypothesis. It will be very interesting and meaningful to further explore the mechanism and physiological roles of BPMs in the auxin signaling pathway.

## Materials and methods

### Plant materials and growth conditions

All Arabidopsis (*Arabidopsis thaliana*) materials used as WT controls and for generating transgenic lines in this study were in the Col-0 background. Seeds were sterilized with 70% ethanol for 5 min, washed 4 times with sterile water, and kept at 4 °C for 5 d in darkness. Subsequently, stratified seeds were sown on half-strength Murashige and Skoog (½ MS) media plates containing 1% sucrose and 0.8% agar (Sigma-Aldrich, A1296), pH 5.7. After sowing, the seeds were exposed to white light for 12 h to stimulate germination and then placed in continuous darkness at 22 °C for the desired time for phenotypic analysis. Seedlings of Arabidopsis or *N. benthamiana* were grown in soil in a growth room at 22 °C under a long-day (16-h light/8-h dark) photoperiod. Arabidopsis plants used for protoplast preparation were grown in soil at 22 °C with a short-day (8-h light/16-h dark) photoperiod for 4 wk.

### Plasmid construction and plant transformation

Deletion *bpm* mutants using CRISPR-Cas9 technology were generated, as described previously (Gao et al. 2016). Two sgRNA sequences targeting each *BPM* gene were synthesized and amplified using *pCBC-DT1DT2* as a template. The PCR product was then cloned into *pHEE401E-mCherry* vector using HIFI DNA assembly reaction (NEB, E5520s). Since previous studies described T-DNA alleles of these genes, we designated our alleles *bpm1-2*, *bpm2-2*, *bmp4-2*, and *bpm5-2* (Julian et al. 2019; Chico et al. 2020). To construct *pBPM1:BPM1-GFP*, *pBPM2:BPM2-GFP*, *pBPM4:BPM4-GFP*, *pBPM5:BPM5-GFP*, and *pIAA10:IAA10-GFP* plasmids, about 2.5 kb promoter fragments along with *BPM* and *IAA10* genomic sequences were cloned into the *pGWB4* vector. For *35S:BPM1*, *35S:BPM2*, *35S:BPM4*, and *35S:BPM5* construction, the coding sequences of *BPM* genes were cloned into the *pEarley104* vector. To construct *35S:IAA10* lines, the coding sequence of *IAA10* with DII degron mutation, SBC motif mutation and DII-SBC mutation were cloned into the *pMDC43* vector. These constructs were transformed into *Agrobacterium tumefaciens* strain GV3101, and the transformed GV3101 cells were used to generate transgenic Arabidopsis plants using the floral dip method (Clough and Bent 1998). Transformants were selected based on their resistance to basta or hygromycin. Homozygous T3 or T4 lines were used in various experiments. The *bpm1,4,5 DR5:GFP* reporter line was generated by crossing *bpm1,4,5* with plants carrying the *DR5:GFP* transgene. Primers used for gene cloning are listed in Supplementary Table S1.

### Phenotypic characterization

Etiolated seedlings grown vertically for the indicated time were used for phenotype characterization. Whole seedlings were photographed using Epson V600 flatbed scanners. Hypocotyl lengths, hook angles, and cotyledon opening angles were measured using ImageJ software. The angle of hook curvature was measured as described earlier (Zadnikova et al. 2010). Cotyledon opening angles were measured, as reported previously (Li et al. 2014). Cotyledons open >30° were counted as “opened.”

### Confocal imaging

To image BPMs-GFP, 40HPG etiolated seedlings were mounted with water and viewed with a Zeiss LSM 880 inverted microscope, and 20× objective was used. A 480 nm laser used for GFP and 561 nm laser used for propidium iodide. Tile scan mode was used for capturing images of whole seedlings. To assess the effects of light, images from 40HPG etiolated seedlings were obtained at Time 0 and after 6 h of light with or without Bortezomib (LC Laboratories, B-1408). For IAA treatment, 40HPG etiolated seedlings were immersed in liquid ½ MS media with 5 *μ*m IAA or control for 4 h, and then images were captured in indicated regions using the same microscope settings. To image the *DR5:GFP* lines, whole etiolated seedlings were used to capture images from apical hooks and hypocotyls at indicated time points. To assess *DR5:GFP* signal in cotyledons, cotyledons were first dissected, mounted with water, and images were captured at indicated time points.

### Hypocotyl segment elongation assay

Hypocotyl segment elongation assays were performed essentially as described earlier (Fendrych et al. 2016; Prigge et al. 2020). The 40HPG etiolated seedlings were dissected using a dissecting microscope with its light source filtered with 6 sheets of green cello film. Roots and cotyledons were excised, and the hypocotyls were transferred to plates containing depletion medium [DM; 10 mm KCl, 1 mm MES pH 6 (KOH), 1.5% phytagel] overlain with a piece of cellophane (PaperMart.com). After 60 min on DM, the hypocotyl segments were transferred to auxin treatment plates (DM plus either 5 *µ*m IAA, 5 *µ*m NAA, or the equivalent amount of ethanol). About 10 hypocotyls were used for each genotype and treatment. Using Epson V600 flat-bed scanners, the plates were scanned at 1,200 dpi every 10 min for 3 h. The segments were measured using a FIJI macro described earlier (Prigge et al. 2020).

### Chemical treatment

Stratified seeds were sown on ½ MS media plates supplemented with 10 *µ*M l-kynurenine (Kyn, Sigma-Aldrich, K8625) or mock (H_2_O). The seeds were exposed to white light for 12 h to stimulate germination and grown vertically for the indicated time for phenotypic analysis.

### Adventitious root and lateral root quantification

Adventitious and lateral root quantifications were performed using 12-d-old etiolated seedlings and 9-d-old light-grown seedlings, respectively. Seedlings were fixed, as described previously (Malamy and Benfey 1997). They were incubated in 0.24 N HCl in 20% methanol at 50 °C for 15 min, followed by transfer to a solution of 7% NaOH and 7% hydroxylamine-HCl in 60% ethanol for another 15 min at room temperature. The samples were then immersed in a series of ethanol solutions (40%, 20%, and 10%) for 5 min each, and they were stored in a solution containing 5% ethanol and 25% glycerol. The prepared samples were mounted in 50% glycerol on glass slides and examined under bright field using a 40×/1.2 NA WI objective with a DIC filter on a Zeiss LSM 880 inverted microscope.

### RNA extraction and RT-qPCR analysis

For auxin treatment, 2DAG etiolated seedlings were transferred into liquid ½ MS media with or without 5 *µ*m IAA, and then wrapped and incubated in the chamber for 4 h. For light treatment, 2DAG etiolated seedlings were transferred into liquid ½ MS media, then wrapped or unwrapped in the growth chamber for 6 h. Seedlings were collected and used for total RNA isolation using Qiagen RNeasy Plant Mini kit. The quality of the total RNA was assessed using a NanoDrop spectrophotometer. Two micrograms of RNA were used for RT-PCR using Thermo Maxima H Minus master mix. RT-qPCR was performed using CFX Opus 384 Real-Time PCR System (Bio-Rad) with SsoAdvanced SYBR mix (Bio-Rad). Primers used for qRT-PCR are listed in Supplementary Table S1.

### Analysis of endogenous IAA

To measure IAA levels in hypocotyls and cotyledons of etiolated seedlings, Col-0 and *bpm1,4,5* plants were sown on ½ MS media and harvested at 1DAG, 2DAG, and 3DAG. The roots were removed, and the hypocotyls and cotyledons dissected and analyzed separately. The extraction, purification, and LC–MS analysis of endogenous IAA, its precursors, and metabolites were carried out, as described previously (Novak et al. 2012). Briefly, ∼5 mg of frozen material per sample was homogenized using a bead mill (27 Hz, 10 min, 4 °C; MixerMill, Retsch GmbH, Haan, Germany) and extracted in 1 mL of 50 mm sodium phosphate buffer containing 1% sodium diethyldithiocarbamate (DEDTCA) and the mixture of ^13^C_6_- or deuterium-labeled internal standards. After centrifugation (14,000 RPM, 15 min, 4 °C), the supernatant was derivatized using cysteamine (0.25 m; pH 8; 1 h; room temperature; Sigma-Aldrich); afterwards, the pH of the sample was adjusted to 2.5 by 3 m HCl and applied on a preconditioned solid-phase extraction column Oasis HLB (30 mg 1 cc; Waters Inc., Milford, MA, USA). After sample loading, the column was rinsed with 1 mL 5% methanol. Compounds of interest were then eluted with 2 mL 80% methanol. Mass spectrometry analysis and quantification were performed by an LC–MS/MS system comprising a 1290 Infinity Binary LC System coupled to a 6495 Triple Quad LC/MS System with Jet Stream and Dual Ion Funnel technologies (Agilent Technologies, Santa Clara, CA, USA).

### Yeast 2-hybrid assay

Yeast 2-hybrid assays were based on the Matchmaker LexA 2-hybrid system (Clontech). *BPM* genes were cloned into the *pGlida* vector. *IAA1*, *IAA3*, *IAA6*, *IAA10*, *IAA11*, *IAA13*, *IAA14*, *IAA15*, *IAA16*, *IAA17*, *IAA18*, *IAA19*, *IAA26*, *IAA27*, *IAA30*, *IAA33*, and *IAA34* genes were cloned into *pB42AD* vector by gateway LR reactions*. IAA5*, *IAA7*, *IAA8*, *IAA12*, *IAA28*, and *IAA31* genes were cloned into *pB42AD* vector by 2 restriction enzymes EcoRI and XhoI. Some of the Aux/IAA constructs were described previously (Calderon Villalobos et al. 2012). Constructs were co-transformed into the yeast strain EGY48, and co-transformation with empty vectors (EVs) were used as negative controls. The presence of the transgenes was confirmed by growth on SD-UHW (SD/-His/-Trp/-Ura) plates. Interactions were observed by detecting β-galactosidase activity on SD/Gal/Raf/X-Gal plates (with 50 *µ*m IAA or Ethanol) after 3 d of incubation at 30 °C. The primers used in this study are listed in Supplementary Table S1.

### BiFC assay

*BPM1-VYNE(R)* was generated by an LR reaction between *pENTR-BPM1* and *VYNE(R)*, while *IAA10-VYCE(R)* was generated via an LR reaction between *pENTR-IAA10* and *VYCE(R)*. A multiple cloning site was cloned into EVs to generate *MCS-VYNE(R)* and *MCS-VYCE(R)* as negative controls. These constructs were transformed into *A. tumefaciens* strain GV3101 and different combinations were co-infiltrated into *N. benthamiana* leaves together with p19 strain. The transformed plants were grown in the dark for 1 d and then transferred to long-day conditions for 2 d. Complemented Venus fluorescence was observed using Zeiss LSM 880 inverted microscope.

### Co-IP, degradation, and ubiquitylation assays in protoplasts

For Co-IP assays, *BPM1* was cloned into *35S:Myc* plasmid, and *IAA10* was cloned into *pJIT163-GFP* plasmid. Arabidopsis mesophyll protoplasts were prepared and transfected, as described previously (Yoo et al. 2007). Protoplasts were transfected with *GFP/Myc*, *GFP/BPM1-Myc*, *IAA10-GFP/Myc*, or *IAA10-GFP/BPM1-Myc*, respectively, and incubated for 16 h. Total protein from protoplast cells was extracted using protein extraction buffer [50 mm Tris-HCl (pH 7.5), 150 mm NaCl, 5 mm EDTA, 1 mm Phenylmethylsulfonyl fluoride, 1 mm dithiothreitol, protease inhibitor cocktail (Roche, 11836170001) and 1% Triton X-100] with 30 *µ*m Bortezomib. After protein extraction, 20 *µ*L of GFP-trap Agarose beads (Fisher Scientific, GTA020) were added to each reaction for 2 h at 4 °C. The precipitated samples were washed 3 times with protein extraction buffer and then eluted by boiling the beads in SDS protein loading buffer for 5 min. Immunoblots were detected with an anti-GFP antibody (1:2,000; Fisher Scientific, A11122) and an anti-Myc antibody (1:2,000; Roche, 11814150001).

For degradation assays, *BPM1* and *IAA10* were cloned into *35S:Myc* plasmid to generate *35S:BPM1-Myc* and *35S:IAA10-Myc* constructs. An EV was used as a negative control. Protoplasts were transfected with *BPM1-Myc/EV*, *IAA10-Myc/EV*, or *BPM1-Myc/IAA10-Myc*, respectively, and incubated for 16 h. Protoplasts were then treated with 5 *μ*m IAA and incubated for an additional 4 h. To detect the transfected proteins, total protein from protoplast cells was extracted using protein extraction buffer. Extracted proteins were separated by SDS–PAGE using 12% polyacrylamide gels, transferred to a nitrocellulose membrane, and detected with anti-Myc antibodies (1:2,000; Roche, 11814150001) and anti-actin antibody (1:2,000; Sigma-Aldrich, A0480). To examine the degradation rate, protoplasts were treated with 50 *μ*m CHX and protein levels were analyzed at the indicated times.

In the ubiquitylation assay, protoplasts were transfected with *EV/EV*, *IAA10-GFP/EV*, or *IAA10-GFP/BPM1-Myc*, respectively, and incubated for 16 h. Protoplasts were then treated with 50 *μ*m Bortezomib and incubated for an additional 6 h. Five hundred microliters of total protein from protoplast cells were extracted using protein extraction buffer. Move 50 *μ*L total protein for input, and add 25 *μ*L equilibrated GFP-Trap magnetic beads (Bulldog Bio, GMA020) to 450 *μ*L protein. After 2 h rotated incubation at 4 °C, the beads were gathered using a magnetic stand, washed 3 times with protein extraction buffer, and then eluted by boiling the beads in SDS protein loading buffer for 5 min. Immunoblots were detected with an anti-GFP antibody (1:2,000; Fisher Scientific, A11122), an anti-ubiquitin antibody (Cell Signaling Tech, 3936T, 1:1,000), and an anti-Myc antibody (1:2,000; Roche, 11814150001).

### Accession numbers

Sequence data from this article can be found in the GenBank/EMBL data libraries under accession numbers: *BPM1* (AT5G19000), *BPM2* (AT3G06190), *BPM3* (AT2G39760), *BPM4* (AT3G03740), *BPM5* (AT5G21010), *BPM6* (AT3G43700), and *IAA10* (AT1G04100).

## Supplementary Material

kiaf155_Supplementary_Data

## Data Availability

The data underlying this article are available in the article and in its online supplementary material.

## Supplementary data

The following materials are available in the online version of this article.

**Supplementary Figure S1.** Schematic diagram of knockout positions of different *bpm* mutants.

**Supplementary Figure S2.** Single *bpm* mutants do not exhibit a mutant seedling phenotype.

**Supplementary Figure S3.**  *35S:BPM* transgenic line phenotypes.

**Supplementary Figure S4.** Light-grown *bpm* mutant phenotypes.

**Supplementary Figure S5.** Effects of light on BPM1, BPM4, and BPM5 protein levels.

**Supplementary Figure S6.** Effects of auxin on BPM1, BPM4, and BPM5 protein levels.

**Supplementary Figure S7.** Hypocotyl segment elongation in response to NAA.

**Supplementary Figure S8.** Auxin biosynthesis, transport, and responsive gene transcription were affected in *bpm* mutants.

**Supplementary Figure S9.** Lateral root development in light-grown seedlings of Col-0 and *bpm* mutants.

**Supplementary Figure S10.** EV control does not interact with Aux/IAA proteins in yeast 2 hybrid assay.

**Supplementary Figure S11.** BPM1 interacts with and recognizes the SBC motif for degradation in IAA10.

**Supplementary Figure S12.** Models of CUL3^BPMs^ regulate the stability of the Aux/IAA proteins and function in seedling development.

**Supplementary Table S1.** List of primers used in this study.

## Dive Curated Terms

The following phenotypic, genotypic, and functional terms are of significance to the work described in this paper:

IAA11 Gramene: AT4G28640
IAA11 Araport: AT4G28640

IAA6 Gramene: AT1G52830
IAA6 Araport: AT1G52830

BPM3 Gramene: AT2G39760
BPM3 Araport: AT2G39760

LAX3 Gramene: AT1G77690
LAX3 Araport: AT1G77690

IAA12 Gramene: AT1G04550
IAA12 Araport: AT1G04550

IAA15 Gramene: AT1G80390
IAA15 Araport: AT1G80390

IAA34 Gramene: AT1G15050
IAA34 Araport: AT1G15050

IAA32 Gramene: AT2G01200
IAA32 Araport: AT2G01200

AUX1 Gramene: At2G38120
AUX1 Araport: At2G38120

IAA13 Gramene: AT2G33310
IAA13 Araport: AT2G33310

IAA14 Gramene: AT4G14550
IAA14 Araport: AT4G14550

IAA18 Gramene: AT1G51950
IAA18 Araport: AT1G51950

IAA19 Gramene: AT3G15540
IAA19 Araport: AT3G15540

IAA28 Gramene: AT5G25890
IAA28 Araport: AT5G25890

BPM1 Gramene: AT5G19000
BPM1 Araport: AT5G19000

BPM2 Gramene: AT3G06190
BPM2 Araport: AT3G06190

BPM4 Gramene: AT3G03740
BPM4 Araport: AT3G03740

BPM5 Gramene: AT5G21010
BPM5 Araport: AT5G21010

BPM6 Gramene: AT3G43700
BPM6 Araport: AT3G43700

IAA10 Gramene: AT1G04100
IAA10 Araport: AT1G04100
