# Supplementary material for: BTB/POZ-MATH proteins regulate Arabidopsis seedling development by promoting auxin-independent degradation of the Aux/IAA protein IAA10
Source: Plant Physiol. 2025 Apr 21;198(1):kiaf155. doi: 10.1093/plphys/kiaf155 (PMC12043071; doi:10.1093/plphys/kiaf155)
Supplement: kiaf155_Supplementary_Data [file kiaf155_supplementary_data.pdf]

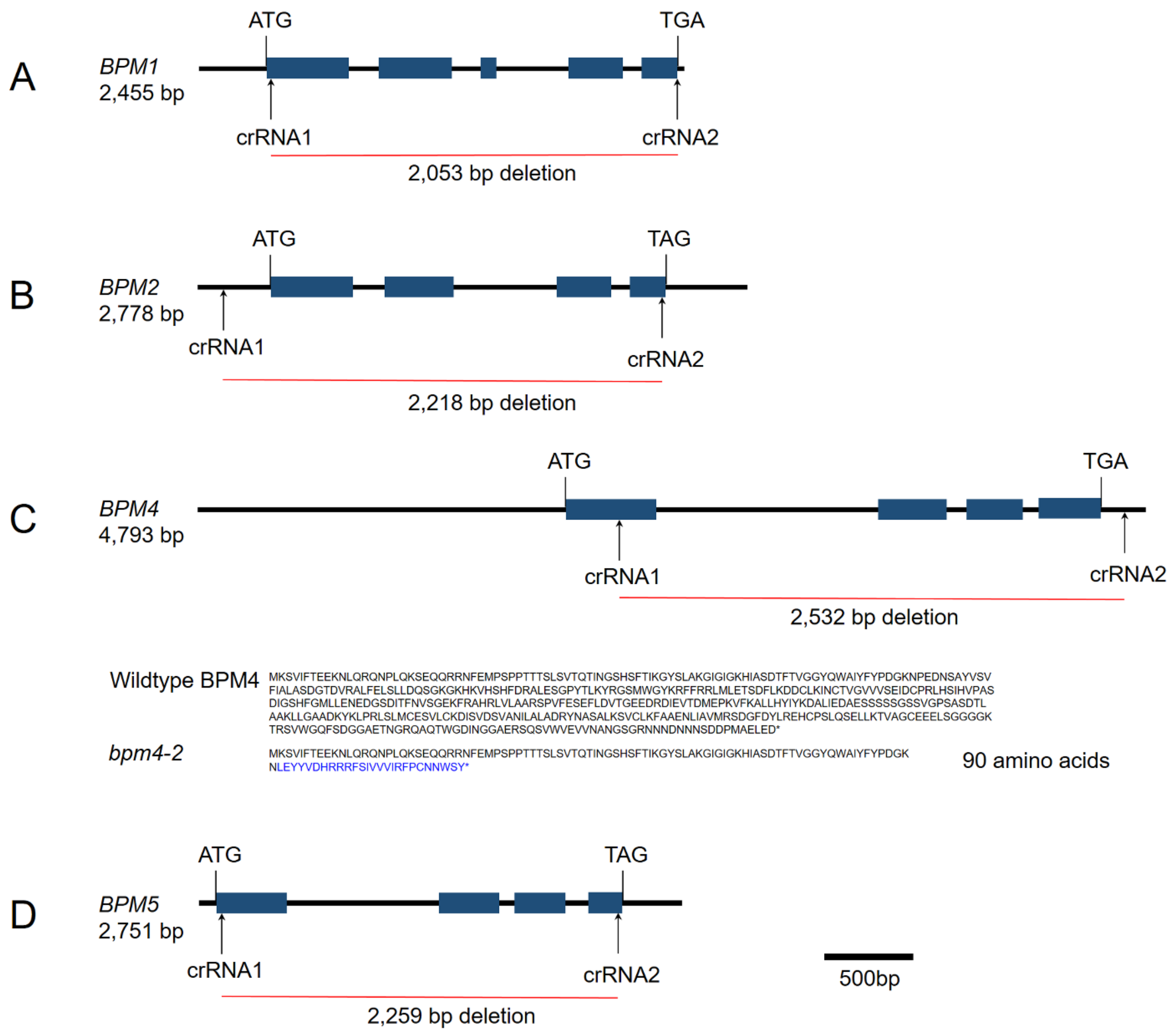

Supplementary Figure S1. Schematic diagram of knockout positions of different *bpm* mutants. Deletion information for four *bpm* mutations created using CRISPR/Cas9 technology. **(A)** *bpm1-2*, **(B)** *bpm2-2*, **(C)** *bpm4-2*, the blue-colored amino acids indicate an incorrect amino acids translation and premature termination in *bpm4-2* mutant. **(D)** *bpm5-2.*

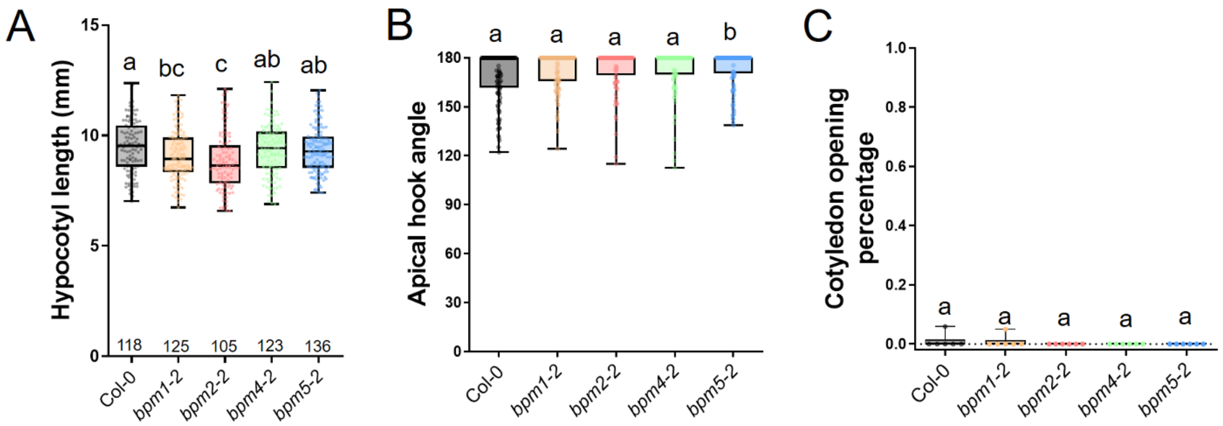

Supplementary Figure S2. Single *bpm* mutants do not exhibit a mutant seedling phenotype. Characterization of 60HPG single *bpm* mutants compared to Col-0. **(A)** Hypocotyl length, **(B)** apical hook angle, **(C)** cotyledon opening percentage. (**B**) and (**C**) use the same sample size as (**A**) in the corresponding lines, and (**C**) was calculated as a percentage in five groups. Boxplots in (**A-C**) represent the median and the first and third quartiles, with whiskers extending to minimum and maximum value; all data points are shown as dots. Statistical differences according to ordinary one-way ANOVA coupled with Holm-Sidak's multiple comparison tests ($P < 0.05$) indicated by letters.

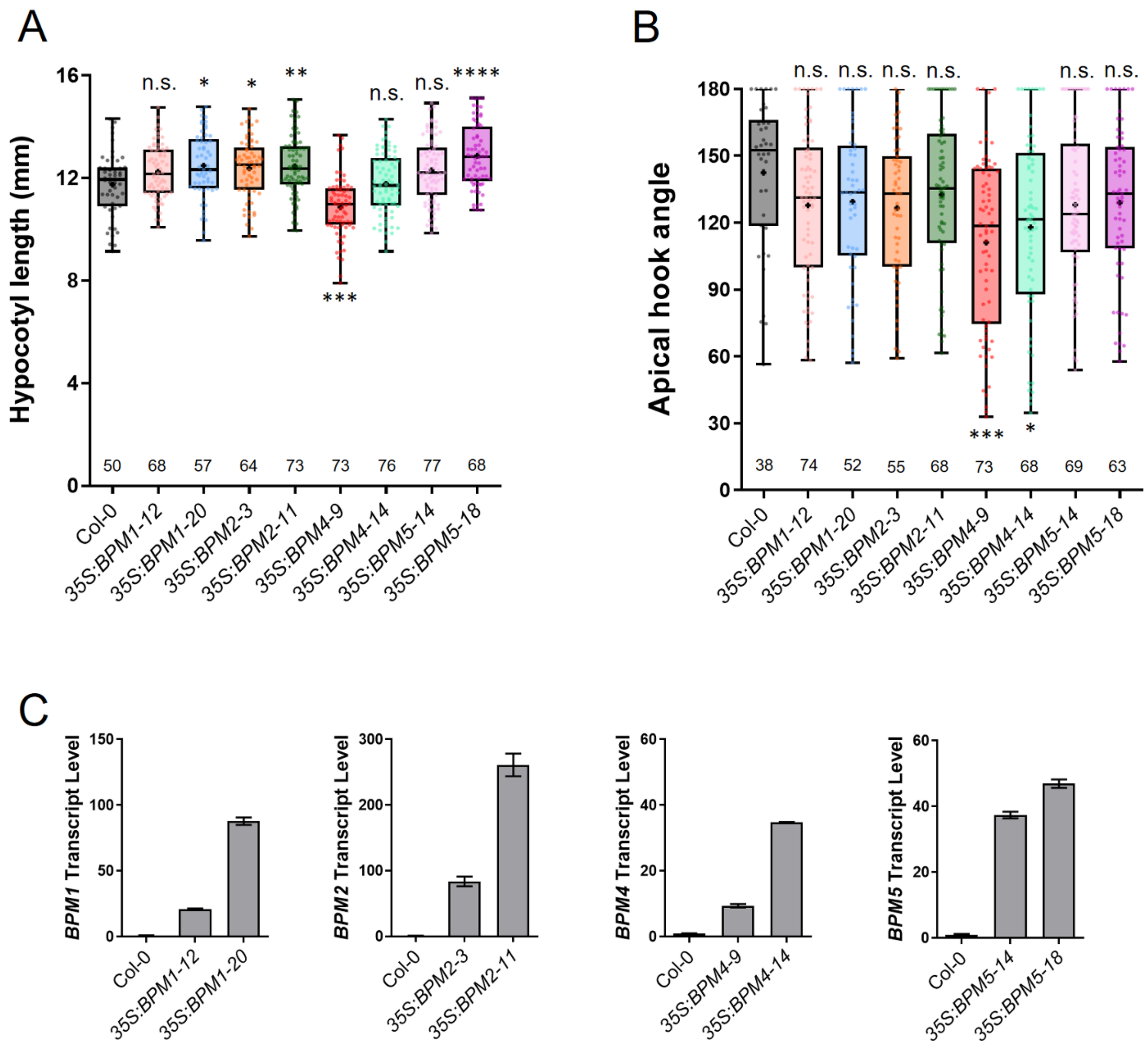

Supplementary Figure S3. *35S:BPM* transgenic lines phenotypes. **A-B)** Analysis of 3DAG *35S:BPM* lines and Col-0. **(A)** Hypocotyl length and **(B)** apical hook angle. Boxplots in (**A-B**) represent the median and the first and third quartiles, with whiskers extending to minimum and maximum value; all data points are shown as dots. Statistical differences according to one-way ANOVA analysis by comparing each genotype to Col-0. *$P < 0.05$, **$P < 0.01$, ***$P < 0.001$, ****$P < 0.0001$. **C)** *BPM1, BPM2, BPM4, BPM5* transcript levels in different *35S:BPM* overexpression lines. Ten seedlings in each line were used in this assay. Data are represented as mean ± SEM.

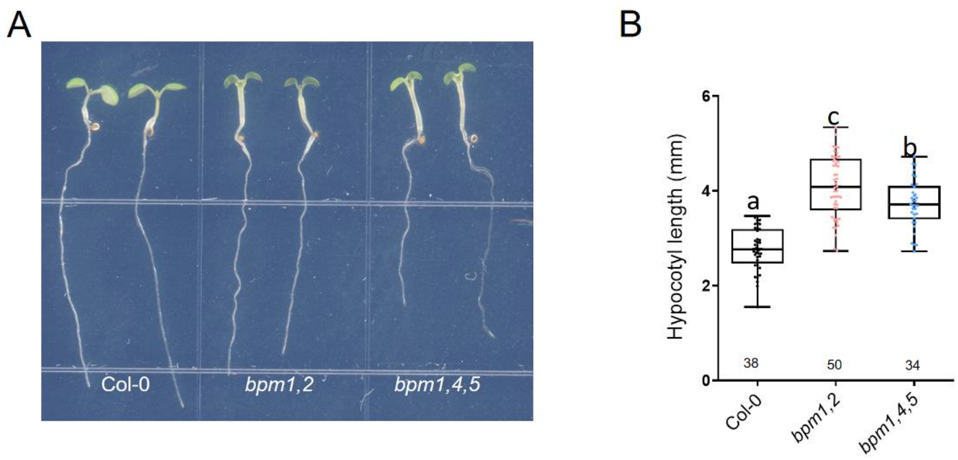

Supplementary Figure S4. Light-grown *bpm* mutants phenotypes. **A)** 7-day old seedlings of Col-0, *bpm1,2* and *bpm1,4,5* under short-day conditions. **B)** Analysis of hypocotyl length in Col-0, *bpm1,2* and *bpm1,4,5.* Boxplots in (**B**) represent the median and the first and third quartiles, with whiskers extending to minimum and maximum value; all data points are shown as dots. Statistical differences according to ordinary one-way ANOVA coupled with Holm-Sidak's multiple comparison tests ($P < 0.05$) indicated by letters.

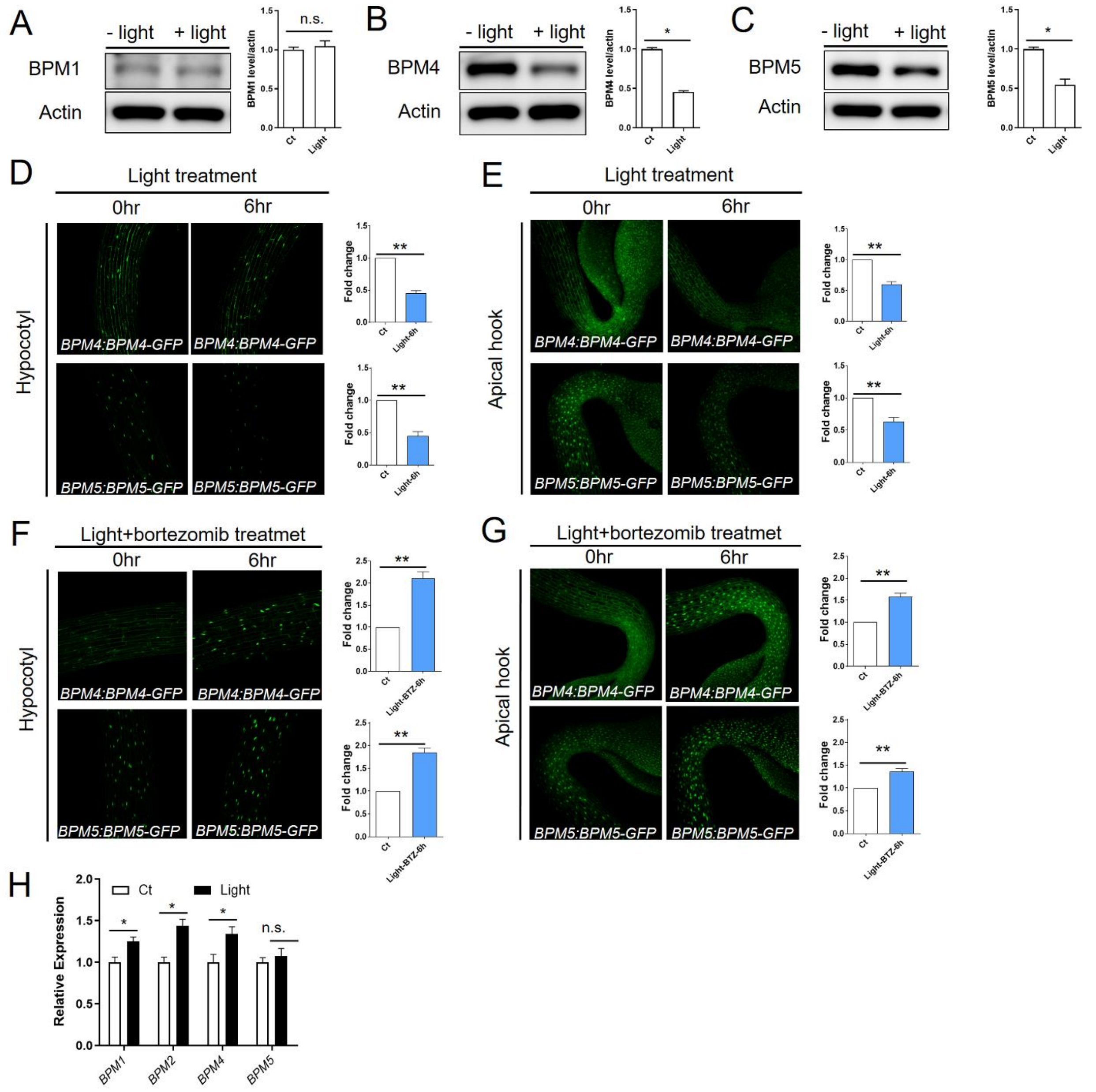

Supplementary Figure S5. Effects of light on BPM1, BPM4, and BPM5 protein levels. **A-C)** Protein levels of BPM1-GFP **(A)**, BPM4-GFP **(B)** and BPM5-GFP **(C)** in native promoter driven transgenic plants under control (constant dark) and 6 hours white light treatment conditions. Three biological replicates were used in (**A**), (**B**), and (**C**). **D-E)** GFP signal in *BPM4-GFP* and *BPM5-GFP* lines after light treatment for 0 hr and 6 hours. The GFP signal in hypocotyls **(D)** and apical hooks **(E)** are shown and quantified. **F-G)** GFP signal in *BPM4-GFP* and *BPM5-GFP* lines after light plus Bortezomib treatment for 0 and 6 hours. The GFP signal in hypocotyls **(F)** and apical hooks **(G)** are shown and quantified. Fifteen seedlings were used for quantification in (**D**), (**E**), (**F**),

and (**G**). **H)** Transcript levels of *BPM* genes under control and light treatment conditions. Expression levels of indicated genes were normalized to *ACTIN7* expression. Light treatment expressions were presented as the fold change to the expression of control. Number of biological replicates = 3. Data are represented as mean ± SEM. (Student's T test, *$P < 0.05$, **$P < 0.01$).

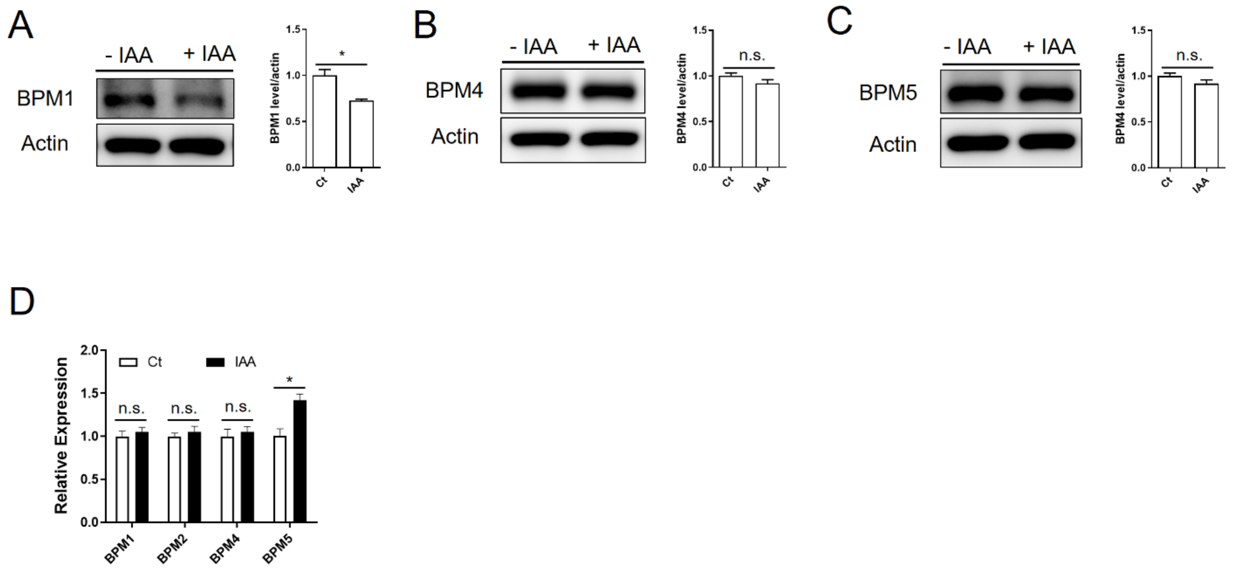

Supplementary Figure S6. Effects of auxin on BPM1, BPM4, and BPM5 protein levels. **A-C)** Protein levels of BPM1-GFP **(A)**, BPM4-GFP **(B)** and BPM5-GFP **(C)** with or without 5 µM IAA treatment for 4 hours. **D)** *BPM* transcript levels with or without 5 µM IAA treatment conditions. Expression levels of indicated genes were normalized to *ACTIN7* expression. The effects of IAA are presented as the fold change relative to the control. Three biological replicates were used in these assays. Data are represented as mean ± SEM. (Student's T test, *$P < 0.05$).

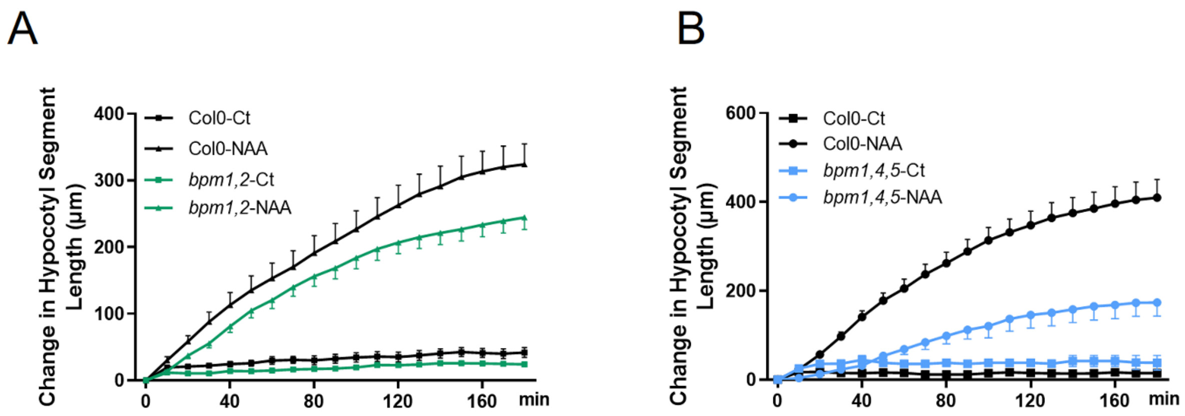

Supplementary Figure S7. Hypocotyl segment elongation in response to NAA. Changes in hypocotyl segment length were quantified in *bpm1,2* **(A)** and *bpm1,4,5* **(B)** and compared to Col-0. 5 µM NAA was used in this assay. Ten seedlings in each line were used in these assays. Error bar indicates ±S.E.M.

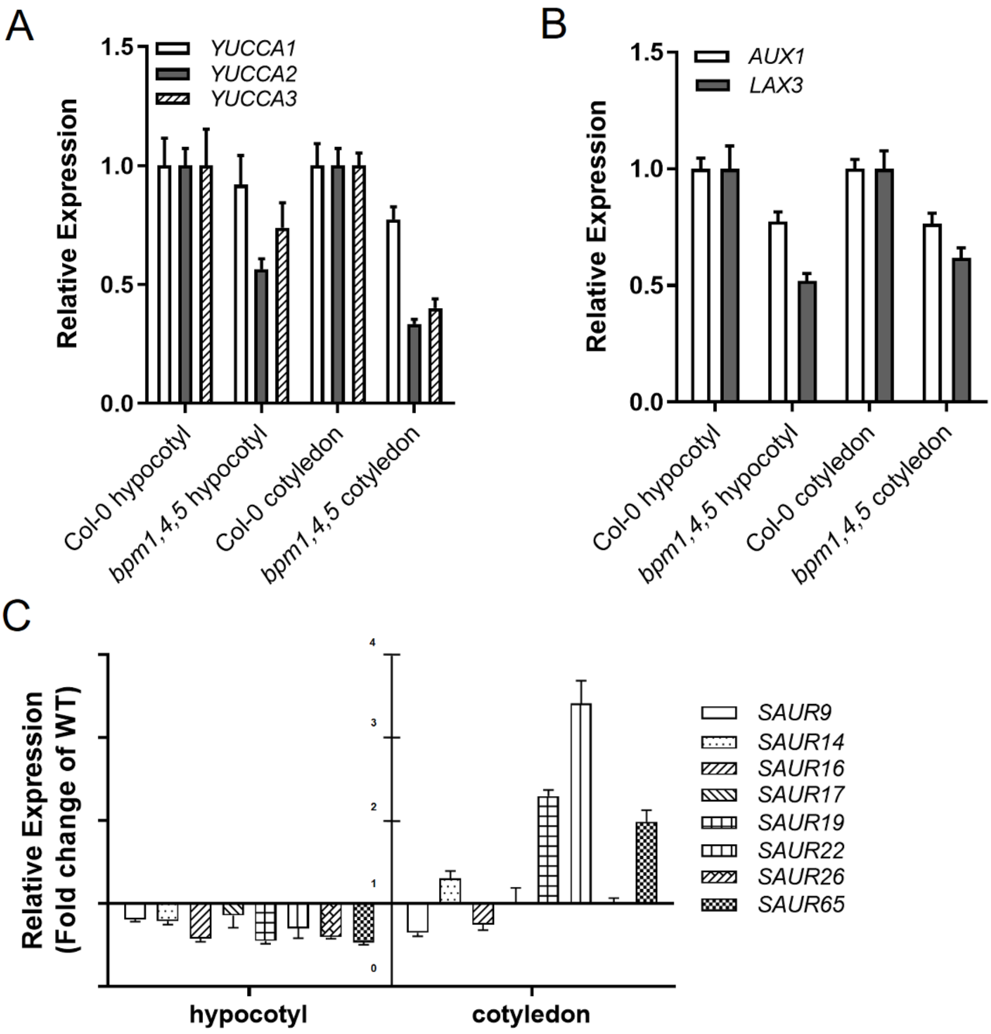

Supplementary Figure S8. Auxin biosynthesis, transport and responsive genes transcription were affected in *bpm* mutants. **A)** Transcript levels of auxin-related genes in Col-0 and *bpm1,4,5.* **B)** Transcript levels of selected *SAUR* genes in hypocotyl and cotyledon in *bpm1,4,5.* The expression levels were presented as the fold change relative to Col-0. **C)** Expression of auxin-responsive genes in Col-0 and *bpm1,4,5* in hypocotyl segments treated with IAA. Three replicates were used in these assays. Data are represented as mean ± SEM.

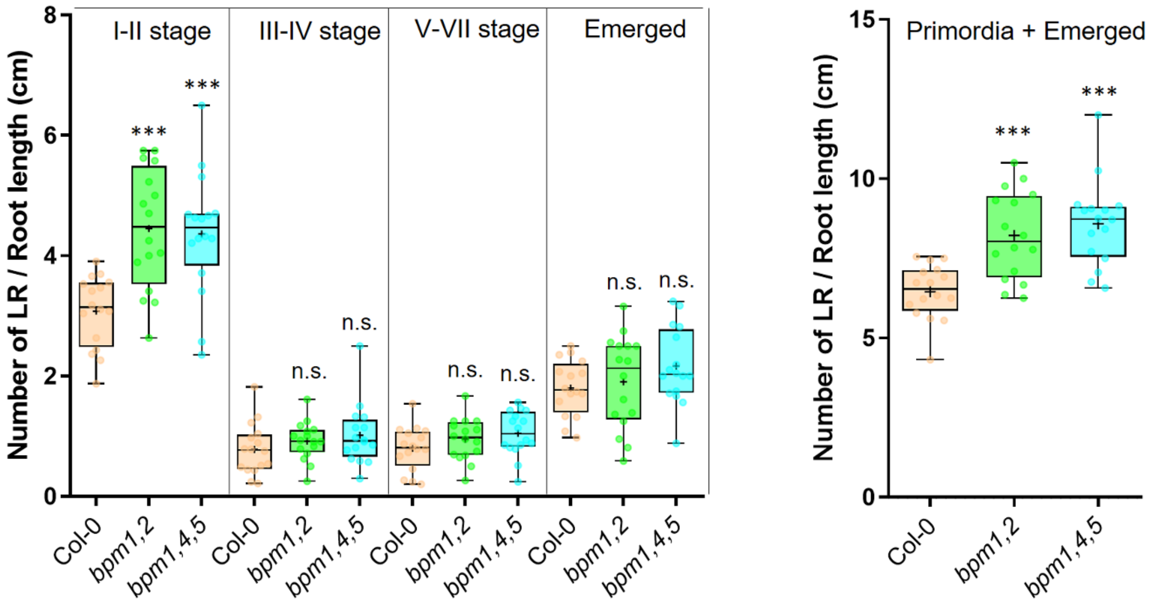

Supplementary Figure S9. Lateral roots development in light-grown seedlings of Col-0 and *bpm* mutants. Lateral root primordia and emerged lateral roots in Col-0 and *bpm* mutants were observed in 9-day-old light-grown seedlings. Sixteen seedlings of each genotype were used for quantification. Boxplots represent the median and the first and third quartiles, with whiskers extending to minimum and maximum value; all data points are shown as dots. Statistical differences according to one-way ANOVA analysis by comparing each genotype to Col-0. *$P < 0.05$, **$P < 0.01$, ***$P < 0.001$.

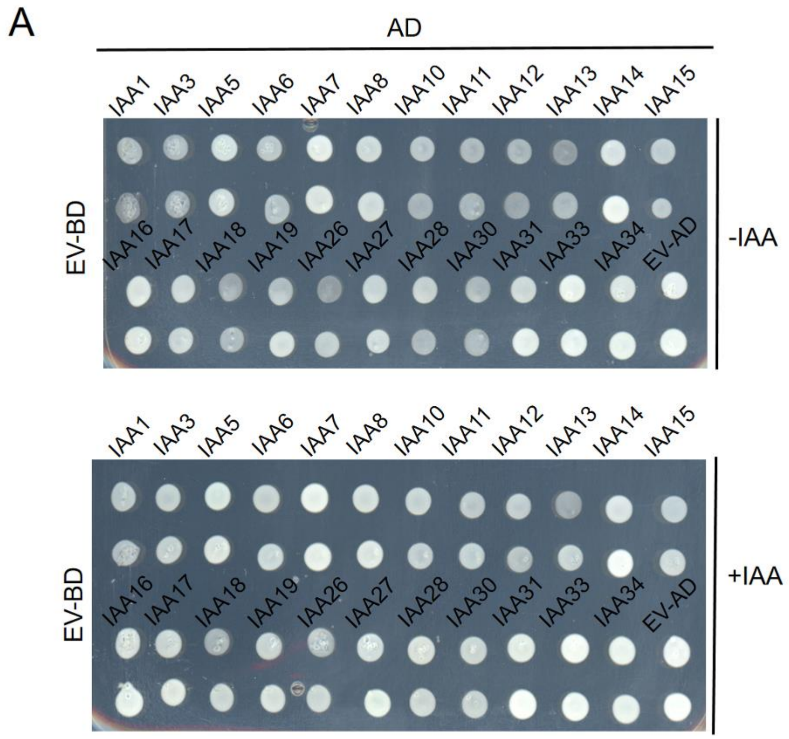

Supplementary Figure S10. Empty vector control does not interact with Aux/IAA proteins in yeast two hybrid assay. Empty vector *pGlilda* was used in BD, and *Aux/IAA* genes were cloned in *pB42AD* vector.

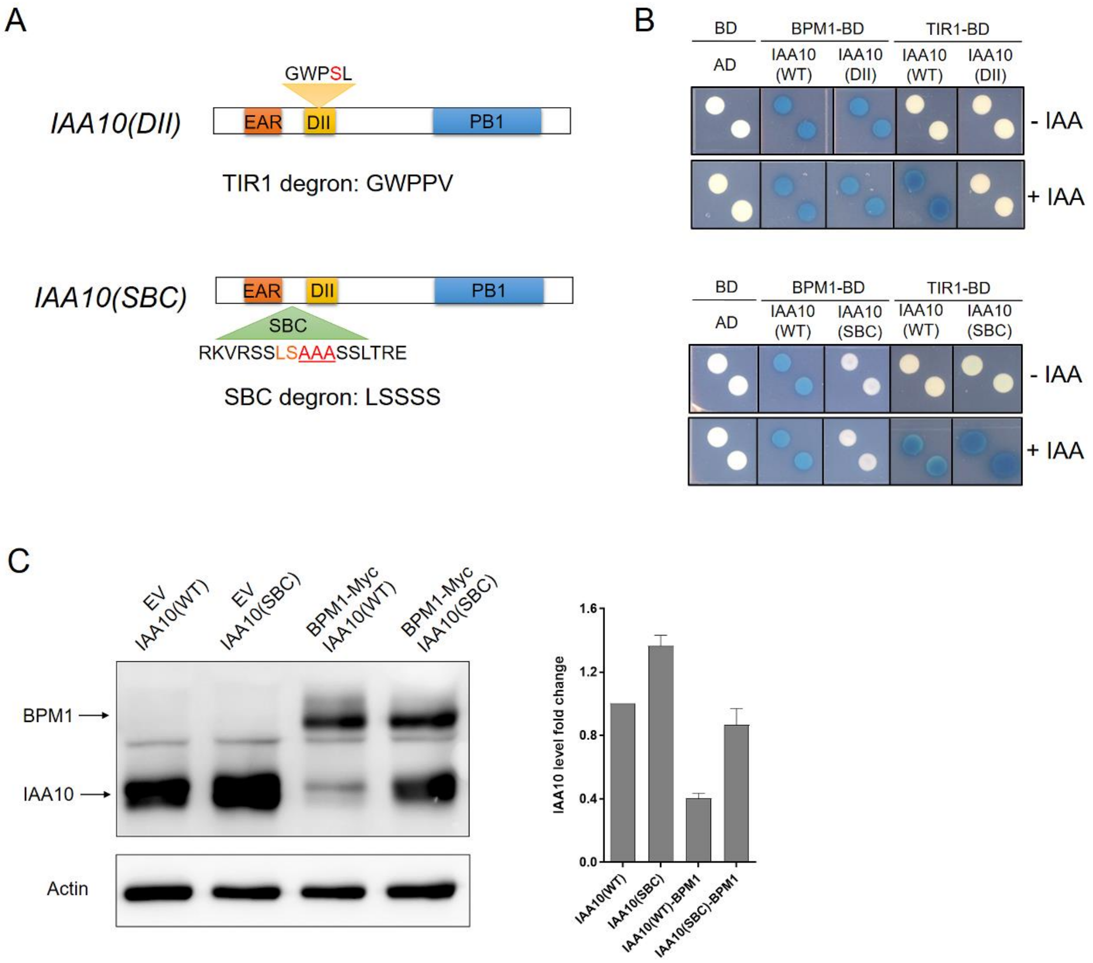

Supplementary Figure S11. BPM1 interacts with and recognizes the SBC motif for degradation in IAA10. **A)** Schematic diagram showing the positions of the DII and SBC degrons in *IAA10*. **B)** Yeast two-hybrid test of BPM1 and TIR1 interaction with different versions of IAA10. IAA10 (WT) refers to wild-type version, IAA10 (DII) stands for DII mutant version, and IAA10 (SBC) stands for SBC mutant version. **C)** Degradation assay with two versions of IAA10 (wild-type and SBC mutant versions) and BPM1 in Arabidopsis protoplasts. Five replicates were used and data are represented as mean ± SEM.

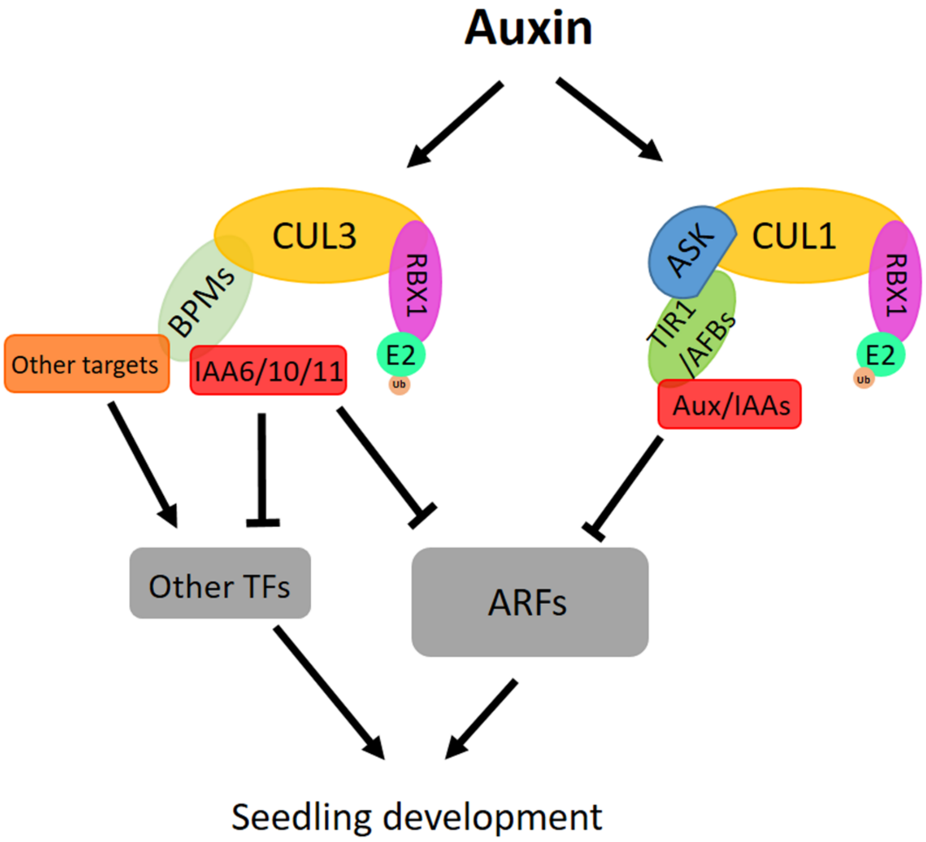

Supplementary Figure S12. Model of CUL3^{BPMs} regulate stability of the Aux/IAA proteins and function in seedling development. In addition to the canonical TIR1/AFBs-Aux/IAAs-ARFs module of the auxin signaling pathway, members of the BPM protein family, which act as substrate adapters for CRL3 E3 ligases, also contribute to auxin signaling by targeting IAA6/10/11 for ubiquitylation and degradation. This process likely releases the repression of ARFs, thereby regulating seedling development. BPMs may also recognize and regulate additional targets involved in this biological process.

Supplementary Table S1. List of primers used in this study.

Name	Sequence	Purpose
BPM1-P1	ctagagtcgaagtagtgattgACAACTAGGGTCTGCTCGGgttttagagctagaaatagc	Crispr line cloning
BPM1-P2	tgctatttctagctctaaaacCGGTTGCACTGAatgggagCAATCTCTTAGTCGACTCTA	Crispr line cloning
BPM2-P1	ctagagtcgaagtagtgattgTGGGAGACGTGTGAAGCAAgttttagagctagaaatagc	Crispr line cloning
BPM2-P2	tgctatttctagctctaaaacgcgattatcggtctctcacCAATCTCTTAGTCGACTCTA	Crispr line cloning
BPM4-P1	ctagagtcgaagtagtgattgtccacatagtattcgacaggttttagagctagaaatagc	Crispr line cloning
BPM4-P2	tgctatttctagctctaaaacCAGGATTCTTGCCATCAGGCAATCTCTTAGTCGACTCTA	Crispr line cloning
BPM5-P1	ctagagtcgaagtagtgattgGTGATTCAGGGATCGAATCgttttagagctagaaatagc	Crispr line cloning
BPM5-P2	tgctatttctagctctaaaacGCCGACATGTTAGACAACGCAATCTCTTAGTCGACTCTA	Crispr line cloning
BPM1-GT1	GGCTACAGCTTCTCTAGCTGCC	Genotyping for Crispr line
BPM1-GT2	AAACGCGTTGAAAACGATTCATTGTAC	Genotyping for Crispr line
BPM1-GT3	GGACCGCTATCGAGAGCTCTACC	Genotyping for Crispr line
BPM2-GT1	GCTAGCTGAACAACACAGATCAACC	Genotyping for Crispr line
BPM2-GT2	ATGAAATTTGCACAGAATAATCTATA	Genotyping for Crispr line
BPM2-GT3	GCTTGCAGCTGCAGACCG	Genotyping for Crispr line
BPM4-GT1	GCTCTTAGTATTTATCCAAGGAGTTAACACTTG	Genotyping for Crispr line

BPM4-GT2	GATACTTTCACCGTCGGGGG	Genotyping for Crispr line
BPM4-GT3	CAGCTGTTATGAGGTCAGATGGGTTTG	Genotyping for Crispr line
BPM5-GT1	CCTCTCTATGGCTACTATTAACCTGAGG	Genotyping for Crispr line
BPM5-GT2	GATCCAATGAGGAAGGATGATGTT	Genotyping for Crispr line
BPM1-F	CACCATGGGCACAACTAGGGTCTG	pENTR
BPM1-R	GTGCAACCGGGGCTTCAC	pENTR (no stop codon)
BPM1-R-stop	TCAGTGCAACCGGGGCTTCAC	pENTR (with stop codon)
BPM2-F	CACCATGGACACAATTAGGGTTTCCAAGGAG	pENTR
BPM2-R	ATGTAACCGTTGCTTCACACGTCTC	pENTR (no stop codon)
BPM2-R-stop	CTAATGTAACCGTTGCTTCACACGTC	pENTR (with stop codon)
BPM4-F	CACCATGAAATCTGTCATTTTCACAGAGGAAAAAAACTT	pENTR
BPM4-R	ATCTTCTAGTTCTGCCATTGGGTCATC	pENTR (no stop codon)
BPM4-R-stop	TCAATCTTCTAGTTCTGCCATTGGGTC	pENTR (with stop codon)
BPM5-F	CACCATGTCAGAATCAGTGATTCAGGGATCG	pENTR
BPM5-R	GGTGGTTCGTTGTCTAACATGTCGG	pENTR (no stop codon)
BPM5-R-stop	CTAGGTGGTTCGTTGTCTAACATGTCG	pENTR (with stop codon)
IAA10-F	CACCATGAATGGTTTGCAAGAAGTTTGTTCGT	pENTR
IAA10-R	CTTACCTACTCCAGCTCCAATTGATGTCTT	pENTR (no stop codon)
IAA10-R-stop	CTACTTACCTACTCCAGCTCCAATTGATGTCTT	pENTR (with stop codon)

pGilda-BPM2-F	CGGGATCCGTATGGACACAATTAGGGTTTCCAAGGAG	Yeast two hybrid
pGilda-BPM2-R	CCGCTCGAGCTAATGTAACCGTTGCTTCACACGTCTC	Yeast two hybrid
pGilda-BPM4-F	CGGGATCCGTATGAAATCTGTCATTTTCACAGAGGAAAAAAAC	Yeast two hybrid
pGilda-BPM4-R	CCGCTCGAGTCAATCTTCTAGTTCTGCCATTGGGTC	Yeast two hybrid
pGilda-BPM5-F	CGGGATCCGTATGTCAGAATCAGTGATTCAGGGATCG	Yeast two hybrid
pGilda-BPM5-R	CCGCTCGAGCTAGGTGGTTCGTTGTCTAACATGTCG	Yeast two hybrid
pGilda-BPM6-F	CGGGATCCGTATGTCAAAGCTAATGACCAGAACCAGC	Yeast two hybrid
pGilda-BPM6-R	CCGCTCGAGCTAAGTGGTTCGCTGCCTGACC	Yeast two hybrid
BPM1-SalI-F	ACGCGTCGACATGGGCACAACTAGGGTCTG	Degradation assay
BPM1-SalI-m-R	ACGCGTCGACTCGTGCAACCGGGGCTT	Degradation assay
IAA10-SalI-F	ACGCGTCGACATGAATGGTTTGCAAGAAGTTTGTTCG	Degradation assay
IAA10-SalI-m-R	ACGCGTCGACTCCTTACCTACTCCAGCTCCAATT	Degradation assay
IAA10-SalI-R	ACGCGTCGACCTTACCTACTCCAGCTCCAATT	Co-IP assay
IAA10-P99S-F	GCTGTAGGTTGGCCGTCTCTACGGACT	IAA10 DII mutation
IAA10-P99S-R	AGTCCGTAGAGACGGCCAACCTACAGC	IAA10 DII mutation
IAA10-AAA-F	CGATCGTCTTTGTCTGCTGCTGCTTCTTCTCTG	IAA10 SBC mutation
IAA10-AAA-R	CAGAGAAGAAGCAGCAGCAGACAAAGACGATCG	IAA10 SBC mutation

Actin7-qF	CCATTCAGGCCGTTCTTTC	qPCR
Actin7-qR	CGTTCTGCGGTAGTGGTGA	qPCR
BPM1-qF	AGCCAATCTCTCACGGTATC	qPCR
BPM1-qR	AACAAAGCCCTAACATCAGC	qPCR
BPM2-qF	CGTGTCTCTCTTCATAGCCC	qPCR
BPM2-qR	TGTCCTTCAGATAGTCCGATG	qPCR
BPM4-qF	GCTCACAGGTTAGTATTGGCTG	qPCR
BPM4-qR	GCACTGACTCGCACATTAGAC	qPCR
BPM5-qF	TTGCTGGCGAGAAGTTTC	qPCR
BPM5-qR	CACAACAACCTGAGCCTAATC	qPCR
SAUR9-qF	TCAACACCGAAGTCGCTATG	qPCR
SAUR9-qR	TCGTGCTCGAAACCAAACTC	qPCR
SAUR14-qF	AAGGCAGAGGAAGAGTTTGG	qPCR
SAUR14-qR	GAAGCGAGAAGCAAGATCAATAAA	qPCR
SAUR16-qF	GCTCGAGCTTAGGCAAGAAA	qPCR
SAUR16-qR	GGATGAGTCAAGAAGGAGATTGG	qPCR
SAUR17-qF	TGACATGCACTTCCACGAAAGAG	qPCR
SAUR17-qR	GTTGTGGGGATCACGAATCTTTC	qPCR
SAUR19-qF	GATTCTAAGCCGCTCCAC	qPCR
SAUR19-qR	CCGAGAAGTCACATTGATG	qPCR
SAUR22-qF	GACAAATAGAGAATTATAAATGGCTCTG	qPCR
SAUR22-qR	ATGAATTAAGTCTATATCTAACTCGGAAA	qPCR
SAUR26-qF	GGCTTTGGTGAGAAGTCTCTTTA	qPCR
SAUR26-qR	CCGACGTACACTGCAAGAAA	qPCR
SAUR65-qF	AACAAAGAGCTGCCCTCAAGA	qPCR
SAUR65-qR	AAACAGCCCTTCTCCACAGC	qPCR
YUCCA1-qF	TTCCTAACGGCTGGAGAGGA	qPCR
YUCCA1-qR	TATTCCTGGTGGACCCCTTG	qPCR
YUCCA2-qF	CCTTCTCTTGTGGTTCGTG	qPCR
YUCCA2-qR	GGAGTCTTTCCGCATTTG	qPCR
YUCCA3-qF	TCTACAATCACGGAGCAAAC	qPCR
YUCCA3-qR	ATAATCCTCGCCAGAAAGAG	qPCR
AUX1-qF	TGGGAGAAAGTGATTGGG	qPCR

AUX1-qR	CACCACGAAAGCATTCAAC	qPCR
LAX3-qF	TGGTTTGAGGTGTTAGATGG	qPCR
LAX3-qR	GTAAGTGGTCATAGCGAGTCC	qPCR
IAA10-qF	GCTTTATCAAATCGTCCAGG	qPCR
IAA10-qR	GTCTTGTATCCTTGTGTGTTGG	qPCR
